# Place-based rural health professional pre-registration education programs: a scoping review

**DOI:** 10.3389/fmed.2025.1546701

**Published:** 2025-08-14

**Authors:** Lara Fuller, Jessica Beattie, Matthew R. McGrail, Vincent L. Versace, Gary D. Rogers

**Affiliations:** ^1^School of Medicine, Deakin University, Geelong, VIC, Australia; ^2^Rural Community Clinical School, School of Medicine, Deakin University, Colac, VIC, Australia; ^3^Rural Clinical School, The University of Queensland, Rockhampton, QLD, Australia; ^4^Deakin Rural Health, School of Medicine, Deakin University, Warrnambool, VIC, Australia

**Keywords:** place-based, social accountability, health professional education, rural workforce development, comprehensive program design, scoping review, rural medical education, widening access

## Abstract

**Introduction:**

With an increasing focus on social accountability in program design in response to a shortage of rural healthcare professionals, emerging approaches in pre-registration health professional education (HPE) offer ‘place-based’ solutions. This review assesses the adoption of these approaches by the international HPE community and describes how programs are designed to recruit and train students ‘in place’.

**Methods:**

Utilizing a global scoping review, a search strategy of relevant HPE databases was developed based on the review’s eligibility criteria and key search terms. Titles and abstracts of all articles were screened against the review’s inclusion criteria, followed by full text review of articles retained. Reporting followed the Preferred Reporting Items for Systematic Reviews and Meta-analysis extension for scoping reviews (PRISMA-ScR) checklist.

**Results:**

Database searches identified 4,215 articles (1,526 duplicates). Title and abstract screening were completed, with 319 retained for full text review. Of these, 138 met the inclusion criteria, with 50 unique HPE programs from 12 countries identified, predominantly from medicine or nursing and midwifery. Programs often had a dual purpose to provide a rural workforce and increase access to HPE for under-represented groups. Recruitment strategies included preferential admission of local students, identifying students with rural or primary care intentions, community involvement in selection, and pre-entry programs. A typology of four training models was identified: short-term rural placements, extended rural placements, rural campuses, and distributed blended learning. Distributed blended learning occurred primarily in nursing and midwifery programs, enabling students to train in their home rural communities. Outcomes evaluated by programs focussed on graduates’ work locations, the effectiveness of widening access measures, and academic results.

**Discussion:**

Despite heterogeneity of design and context, place-based programs were characterized by three common features closely aligned with social accountability: widening access to HPE, comprehensive program design and a community-engaged approach. Key considerations for place-based HPE program design are the geographical scale of the program, strategies for student recruitment from the target region, provision of continuity with rural communities through longitudinal training experiences, engaging communities in the design and delivery of the program, and alignment of evaluation with the goals of the program and the communities served.

## Introduction

A shortage of rural healthcare professionals is a global phenomenon that crosses economic, geographic, and health professional discipline boundaries (1–5). There is a need to identify and adopt evidence-based strategies to address this challenge, including through health professional education (HPE) program design (1, 6, 7). Universities act as gatekeepers to the majority of HPE pre-registration training (undergraduate and post-graduate pathways prior to professional registration) and play a critical role in determining who is selected by these programs. High levels of competition for course places and an emphasis on academic attainment favor students of metropolitan origin, largely due to their relative educational, resource and socio-economic advantage (8, 9). Traditionally, universities have also been based in more populated settings, thus reducing access for rural-based applicants, although there have been deliberate policy initiatives this century, such as Australia’s Rural Health Multidisciplinary Training (RHMT) Program, supporting increased rurally-based health professional training (10, 11).

In such an environment, the focus of rural workforce recruitment has often been, understandably, on ‘attract and retain’, with a myriad of strategies focused on enticing a population of predominantly metropolitan based, and trained, graduates into rural practice (1, 12). At the pre-registration level, such strategies have included mandatory periods of rural training, bonded return of service schemes, financial incentives linked to rural practice, and rural curricula (1, 4, 5, 7). In Australia, targeted recruitment of rural background students into pre-registration HPE (particularly in medicine) has been a longstanding strategy aiming to improve rural workforce outcomes, yet this measure alone has not been sufficient to address the maldistribution in that country (13).

There is a substantial body of empirical research that provides guidance on the individual factors associated with enhanced rural health workforce outcomes such as rural origin and longitudinal rural training (14–18). However, there is less evidence available to inform how these individual variables might interact within a complex system, or how programs have been designed and evaluated that incorporate multiple elements. The 2021 WHO guidelines on rural and remote health workforce development included a recommendation for ‘bundling’ of initiatives, as a more effective strategy than isolated interventions (1). Similarly, within medical education there has been increasing discourse on the importance of comprehensive approaches to program design, where several design elements are included (such as rural student selection, extended rural training, and rural curricula) with the expectation of an enhanced outcome (6, 19–21).

The role and importance of social accountability in HPE has gained prominence since the beginning of the 21st century (22–24). Social accountability of educational institutions is defined as ‘the obligation to direct their education, research and service activities toward addressing the priority health concerns of the community, region, or nation that they have a mandate to serve’ (25). The increasing focus on social accountability is reflected in the strengthened recommendations in the 2021 WHO guidelines. Notably, they state that all health workforce education institutions are ‘obliged’ to adopt social accountability as a core part of their mandate to develop a relevant and appropriate workforce (1).

With an increasing focus on both social accountability and comprehensive program design, emerging alternatives to ‘attract and retain’ in HPE have been described as ‘growing your own’ or ‘place-based’ solutions (26–29). These two phrases convey different emphases but share an overlapping purpose to recruit and train students *from* and *for* a specific place. ‘Growing your own’ emphasizes the recruitment of students already embedded in rural communities and providing them with access to training opportunities, ideally in the local area (26, 27). ‘Place-based’ approaches recognize that education is inexorably grounded in the place and context in which it occurs (29). Place-based education (PBE) seeks to strengthen students’ connections with their location and community through the context, content, and pedagogy of curriculum delivery (30–32).

The purpose of this scoping review is to assess the extent of adoption of these approaches by the international HPE community and describe how programs are being comprehensively designed to achieve these goals. A synthesis of the literature on program design in place-based HPE will provide a summary of this evolving field, that will inform contemporary program design and evaluation. Following a search of completed and in-progress reviews, including contacting authors of two reviews in progress on rural PBE, it was determined that the proposed review was substantially different in focus.

## Objectives

This scoping review will identify rural pre-registration HPE programs in the international literature that are adopting a place-based approach to recruiting and training students in a defined geographic region. The review will synthesize the literature to answer the following research questions:

1. What is the global distribution of rural place-based pre-registration HPE programs and how is their concept and purpose described?
2. How is this concept translated into HPE program design?

- How is place defined geographically?
- How is place-based student recruitment occurring?
- How are place-based training and recruitment co-occurring?
- What program outcomes are being investigated?

## Methods

This scoping review was conducted using Arksey and O’Malley’s scoping review guidelines (33) and follows the Preferred Reporting Items for Systematic Reviews and Meta-analysis extension for scoping reviews checklist (PRISMA-ScR) (34) (Supplementary file 1). The review protocol was registered with Open Science Framework (osf.io/56dcf) on 29th May 2024.

### Inclusion criteria

The population, context, and concept framework was used to construct the review’s inclusion criteria (Table 1).

**Table 1:** Inclusion criteria and exclusion criteria.

	Include	Exclude
Population	Must include **both** of the following: health professional education (HPE) program: e.g. medicine, nursing, dentistry, allied health - all types **AND** primary HPE: entry to practice degree (either undergraduate or graduate entry)	non-HPE training programs post-graduate training (beyond entry to professional qualification)
Context	rural/remote/non-metropolitan setting (as stated or defined in the study)	metropolitan setting or setting not stated
Concept	Includes each of the following elements: identifies a focus on a rural region at a scale smaller than national (e.g. region, state, province, territory, town) **AND** recruits students from this region **AND** provides some element of training in the same rural region	student selection from a region but not linked with training in the same region training in a region but not linked with student selection from the region neither student selection nor training linked to a region distance education on a national scale

No restrictions were placed on the type of evidence sources considered for inclusion, with evidence syntheses, editorials and conference presentations considered along with all types of original research study design. There were no restrictions placed on publication date, to allow for a historical perspective on this topic. Only articles available in English were included.

### Information sources

Databases were chosen due to their relevance to HPE: Medline, Cumulative Index of Nursing and Allied Health Literature (CINAHL) and ERIC (Ebscohost), Scopus, and Excerpta Medica database (Embase). Due to the large number of papers identified for screening by the primary database search, the pragmatic decision was taken not to widen the search to grey literature, snowball or hand searching of references, to keep the review size manageable.

### Search strategy

A search strategy was developed based on the review’s eligibility criteria and key search terms identified in papers known to the authors. Search terms were developed using relevant index search terms, keywords, and truncation symbols translated to each individual database and searched separately (Supplementary file 2). Search terms relating to the allied health professions were developed based on terminology used by representative bodies of allied HPE in Australia, United States of America, United Kingdom, and peer-reviewed literature.

Several reference papers known to the authors were used to validate the search strategy. The search strategy was trialed and refined to extract relevant papers from the literature.

### Study selection and data charting

Citations of all articles identified by the database search were exported to Endnote 21 reference library (Clarivate™) and then imported to Covidence^TM^ (Veritas Health Innovation, Melbourne, Australia) for screening. Duplicates were identified and removed by Covidence software, and any remaining duplicates were manually removed during the screening process. Two reviewers (LF and JB) independently screened the title and abstract of all articles against the review’s inclusion criteria. Full text review was then performed independently by the same two reviewers, with reasons provided when articles did not meet the review’s inclusion criteria. Conflicts were resolved by discussion between JB and LF periodically during the screening process.

A data extraction template and guidance document were developed and piloted by two authors (LF and JB), following which data were charted for each included article (LF and JB). Categorical outcomes for extracted data where relevant, were determined during the trialing phase. Data charted by each reviewer were compared during the synthesis of the results, with discrepancies resolved by referring to the primary information source (LF).

For evidence sources that included both rural and metropolitan programs, only rural program information was charted. For literature such as reviews that included multiple programs, only data for programs that met the review’s inclusion criteria were charted. First or corresponding authors were contacted during full text review if additional information was required to determine whether programs met the review’s inclusion criteria. Quality appraisal of articles was not undertaken, consistent with scoping review methodology (33).

### Data items

The following data were extracted to answer the review’s questions:

**Population:** program name, program provider, program description, year of commencement, health profession, entry level (undergraduate/post-graduate), return of service requirements.

**Context:** country, state/province/location, rurality classification of program.

**Concept:** terminology used to describe program’s concept, program purpose, place definition, student selection/recruitment methods, rural training delivery (duration, context, clerkship model), outcome measures.

Metadata of included studies reported included authors, evidence source and year of publication.

## Results

**Figure 1:**
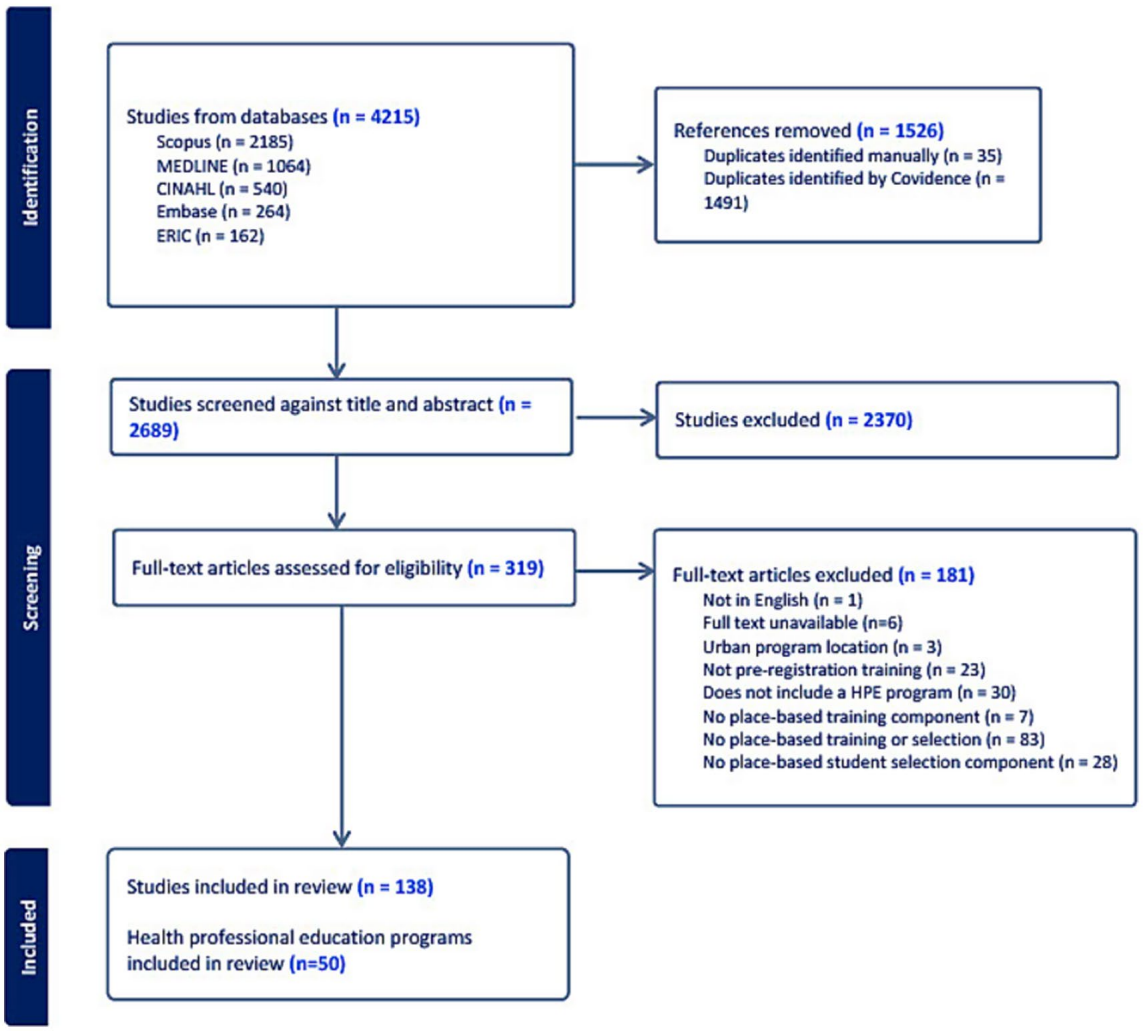
PRISMA-ScR flow diagram.

Database searches conducted on 4th October 2023, identified 4,215 articles. Following the removal of 1,526 duplicates, 2,689 studies were screened by title and abstract against the inclusion criteria, with 2,370 excluded at this stage. Full text review of 319 articles was completed, with 181 studies excluded for the following reasons: no place-based training or selection (*n* = 83), no place-based selection (*n* = 28), not a HPE program (*n* = 30), not pre-registration training (*n* = 23), no place-based training component (*n* = 7), full text unavailable (*n* = 6), urban program location (*n* = 3), not available in English (*n* = 1) (Figure 1 PRISMA-ScR flow diagram).

One hundred thirty-eight information sources met the review’s inclusion criteria, relating to 50 unique programs. Information sources included journal articles (*n* = 129), organizational reports (*n* = 4), symposium/conference proceedings (*n* = 2), a thesis (*n* = 1), a book chapter (*n* = 1), and a WHO bulletin (*n* = 1).

### Research question 1: What is the global distribution of rural place-based pre-registration HPE programs and how is their concept and purpose described?

#### Global distribution

**Figure 2:**
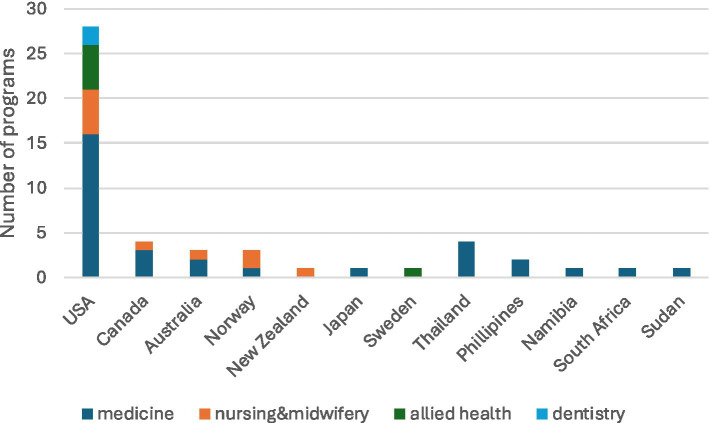
Geographical distribution of health professional education programs.

Of the 50 included programs across 12 different countries, 28 were in the USA, with smaller numbers in Canada (*n* = 4), Thailand (*n* = 4), Australia (*n* = 3), Norway (*n* = 3), the Philippines (*n* = 2) and single programs in Japan, New Zealand, Sweden, South Africa, Sudan and Namibia (Figure 2). Programs were included from seven high income countries (*n* = 41) and five low and middle income countries (*n* = 9) (35) and from five of the six WHO regions, the Eastern Mediterranean Region being the exception (36). Forty programs related to a single educational institution (27 medicine, 9 nursing, 1 dental, 3 allied health), six to a collaborative of more than one institution (1 medicine, 1 nursing, 1 dental, 3 allied health) and four to national level government initiatives (medicine).

**Figure 3:**
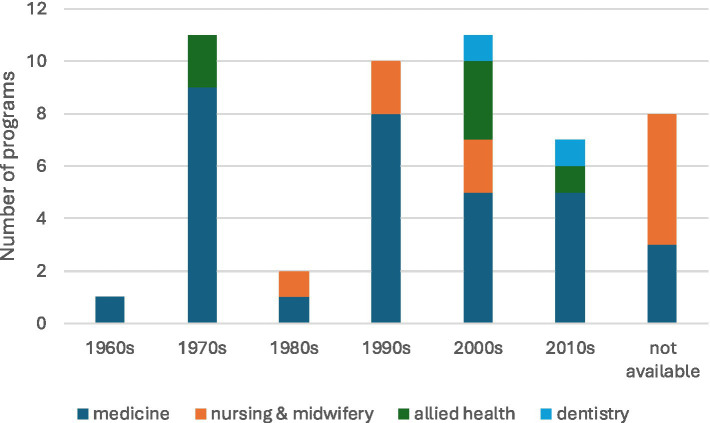
Decade of program commencement by health profession.

The commencement dates of programs ranged from 1967 to 2017. Programs in medicine preceded programs in the other health professional disciplines (Figure 3). Programs are listed in order of commencement date in Table 2 (medical programs) and Table 3 (non-medical programs).

**Table 2:** Characteristics of included medical programs, by year of commencement.

**Program name, location**	**Year of commencement, program description**	**Purpose**	**Place definition**	**Place-based selection methods**	**Place-based rural training**	**Evidence sources/Citations**
Memorial University of Newfoundland and Labrador (MUN), Canada.	1967, 4-year graduate entry.	To meet the workforce needs of the province.	Rural areas of Newfoundland and Labrador province. 52% rural province based on Statistics Canada Subdivision (<10,000).	Reserved places for residents of province. Holistic admissions process to recruit Aboriginal, rural and remote and economically disadvantaged students.	Recurrent short rural clinical placements years 1-4 (2-4 weeks each) and longitudinal rural rotation (3 months).	Tesson et al. (66), Mathews et al. (133), Curran et al. (111), Mathews et al. (134), Rourke (22), Strasser et al. (64), Rourke et al. (58, 99, 106), Grierson et al. (159)
Regional quota scheme (chikiwaku) and prefecture scholarship schemes (both national) Jichi Medical University, Shimotsuke and Asahikawa Medical University, Asahikawa, Japan	1972, 6-year undergraduate.	To produce rural doctors and distribute them nationwide.	Prefecture. Rural area defined by municipality with either <5 physicians OR physician/population ration of 50/100000 or less OR population <20,000 and physician/population ratio <100/100,000.	Admission quota from each prefecture.	Some weeks in home rural prefectures in year 5. Elective rural internship in year 6.	Hsueh et al. (87), Kataoka et al. (71, 72), Kawamoto et al. (51), Matsumoto et al. (45, 165), Yoshida et al. (164), Ozeki et al. (40), Figueiredo et al. (5)
University of Tromso Medical School, Tromso, Norway	1972, undergraduate.	To train graduates preferring to work in rural areas of Northern Norway.	Rural areas of Northern Norway (region). Population density 4/km^2^.	Admission quota from northern Norway, 50%.	Rural training duration/timing not specified.	Magnus et al. (48), Hsueh et al. (87)
Rural Physician Associate Program (RPAP), University of Minnesota Medical School, Duluth, Minnesota, USA	1972, 4-year graduate entry Doctor of Medicine (MD).	To train physicians serving rural Minnesota, emphasizing family medicine and American Indian communities.	Rural areas of Minnesota (state). Internal classifications used for rural Minnesota preceptorships defined as communities with populations less than 20,000 outside of major urban centres.	Preferential admission of students with personal and background traits indicating a high potential for becoming a family physician in a small town, rural setting.	Students live for 3 days each quarter with a rural family medicine preceptor during 2 pre-clinical years, and complete an extended rural community-based placement in year 3 (9 months).	Verby et al. (157), Lampert (105), Keyes-Welch et al. (110), Rabinowitz et al. (156), Geyman et al. (77), Hsueh et al. (87), Crump et al., (39), Rabinowitz et al. (19), Fuglestad et al. (135), Figueiredo et al. (5)
Physician Shortage Area Program (PSAP), Jefferson Medical College, Philadelphia, USA	1974, 4-year graduate entry MD.	To increase the number of family physicians in rural and underserved areas of Pennsylvania.	Rural areas of Pennsylvania (state).	Preferential admission of applicants from rural areas who are committed to rural and family medicine practice. Cooperative arrangements with 6 undergraduate institutions.	Short rural clinical placements, 6 weeks in years 3 and 4 weeks in year 4.	Rabinowitz et al. (74), Rabinowitz et al. (136), Rosenthal (154), Rabinowitz et al. (137), Keyes-Welch (110), Geyman et al. (77), Rabinowitz et al. (156), Hsueh et al. (87), Crump et al. (39), Rabinowitz et al. (138), Rabinowitz et al. (19), Rabinowitz et al. (160), Figueiredo et al. (5)
Upper Peninsula Program (UPP), Michigan State University college of Human Medicine, Michigan, USA	1974, 4-year graduate entry MD.	To improve the physician supply in rural areas of Michigan.	Rural areas of Michigan (state). Distance from main campus 400 miles.	Preferential admission of rural students of Michigan expressing an interest in primary care.	2 years of urban pre-clinical training followed by relocation to one of six community-based campuses for 2 years of rural clinical training. Includes continuous ambulatory training experience in family medicine.	Brazeau et al. (101), Rosenthal et al. (154), Crump et al (39), Hsueh et al., (87), Rabinowitz et al. (156), Rabinowitz et al. (19)
University of Gezira, Faculty of Medicine, Gezira State, Sudan	1975, 5-year undergraduate, community-oriented program.	To meet the workforce needs of rural underserved areas in Gezira region.	Rural areas of Gezira (state).	50% of places reserved for students from underprivileged deprived areas of Gezira State.	25% curriculum community based.	Larkins et al. (100, 141), Woolley (125), Johnston et al. (75)
School of Health Sciences, University of Philippines Manila (UPM-SHS), Palo, Leyte, Philippines	1976, 5-year post-graduate MD.	To meet the needs of rural underserved populations in central Philippines archipelago and Indigenous peoples.	Central Philippines archipelago (region). Population density 358/km^2^.	Students are nominated and endorsed by communities. Preferential selection from lower socio-economic strata and rural and remote communities.	6-month rural community placement in year 2 and community internship in final year (12 months).	Paul (62), Siega-Sur et al. (103), Reeve et al. (76), Woolley et al. (117, 119), Larkins et al. (141), Johnston et al. (75)
Medical Education for Students from Rural Areas Project (MESRAP) program, Chulalongkorn University, Thailand	1978, 6-year undergraduate program.	Community-oriented curricula track to meet the need for more physicians in rural areas.	Province.	Candidate should have lived in the rural province for 5 years, they are identified 2 years before medical school entry, posted to work in provincial hospital, evaluated by staff and short listed for application.	First 3 years at urban campus with community experience during years 2-6 for 3–12-week rotations. 3 clinical years based at provincial hospitals.	Suwanwela et al. (183) Paul (62), Sirisup (146)
West Virginia School of Osteopathic Medicine (WVSOM), West Virginia, USA	1978, 4-year graduate entry, Doctor of Osteopathic Medicine (DO).	To provide primary care physicians for rural Appalachia.	Rural Appalachia (region).	Consortium with other rural Appalachian states. Select students from rural Appalachia (live outside of a Metropolitan Statistical Area (MSA) or in a small town within an MSA) or those motivated to practice there.	Medical school located rurally, fully rural-based program.	Roberts et al. (104), Geyman et al. (77)
Walter Sisulu Medical School (WSU), Faculty of Health Sciences, South Africa	1985, 6-year undergraduate program.	To meet the workforce needs of rural underserved areas of Eastern Cape and KwaZulu natal provinces.	Eastern Cape and KwaZulu natal provinces. Population density 48/km^2^.	Quota system to support Indigenous African enrolments (80%) and those from rural Eastern Cape and KwaZulu Natal provinces (75%).	Rural experiences in years 1-3 and extended placement in year 5 (6 months).	Celletti et al. (158), Larkins et al. (100, 141), Reeve et al. (76), Rourke et al. (58, 99, 106), Johnston et al. (75)
Rural Medical Education track (RMED), University of Illinois College of Medicine, Rockford, Illinois, USA	1993, 4-year graduate entry MD.	To reduce health disparities through producing family physicians for rural Illinois.	Rural areas of Illinois (state). 84 rural counties (population<60,000) in Illinois.	Applicants selected based on rural background, leadership experience, community involvement, and expressed commitment to rural primary care.	Required rural community-oriented clerkship in year 4 based in primary care (4 months).	Keyes-Welch et al. (110), Stearns et al. (21, 61), Rabinowitz (19, 156), Crump et al. (39), Hsueh et al. (87), Glasser et al. (20), Soliman et al. (97), Figueiredo (5)
Ateneo de Zamboanga University School of Medicine (ADZU-SOM), Zamboanga, Philippines	1994, 4-year graduate entry MD.	To meet the needs of rural and underserved populations.	Mindanao in Southern Philippines; especially Zamboanga peninsular and outlying islands. Population density 358/km^2^.	Select local applicants from lower economic strata in Western Mindanao province.	Years 1-3: 1 month each semester living in small rural communities, year 4: entire year in rural community program.	Strasser (152), Larkins et al. (100, 141, 144), Cristobal et al. (93), Halili et al. (118), Reeve et al. (76), Rourke (58, 99, 106), Woolley et al. (117, 119), Johnston et al. (75)
Collaborative Project to Increase Production of Rural Doctors (CPIRD) program, Ministry of Public Health (MOPH), Thailand.	1994, 6-year undergraduate program.	National initiative to increase the production of doctors for rural and remote areas.	Province.	Students are recruited from high schools in rural domiciles in provincial areas.	Urban pre-clinical training followed by 3 years of clinical training outside major cities and near their hometowns.	Putthasri et al. (107), Thammatacharee et al. (121), Nimkuntod et al. (120), Arora et al. (92), Techakehakij et al. (96, 130), Somporn (60, 182), Figueiredo et al. (5)
Rural Track Pipeline Program (MU-RTPP), University of Missouri School of Medicine, Missouri, USA	1995, 4-year Graduate entry MD.	To increase the supply and retention of physicians in rural and underserved Missouri.	Rural areas of Missouri (state). Town<50000 considered rural by program.	Selected from ‘Rural scholars’ pre-admission program. Requires Missouri residence, graduation from a rural high school.	4-8 week summer community program between first and second year, 6-month rural track clerkship (year 3), 1-month rural track elective program (year 4).	Crump et al. 2004 (39), Quinn et al. (56)
Rural Medical Scholars Program (RMSP), University of Alabama, Alabama, USA	1996, 4-year graduate entry MD.	To demonstrate increased production of rural physicians by admitting rural students and emphasizing family medicine.	Rural areas of Alabama (state).	Students must demonstrate 8 years of residence in rural Alabama and have generalist aspirations.	Third-year clerkships in rural family and community medicine.	Wheat et al. (44, 142)
Scholars in Primary Care Program (SPCP), University of Kansas School of Medicine, Kansas, USA	1997, 4-year graduate entry MD.	To increase the number of students who choose to practice primary care medicine in rural Kansas.	Rural Kansas (state). 88 of 105 Kansas counties classified as rural.	Regional quota system for graduates from Kansas rural high schools and College/Universities.	Longitudinal rural track planned for year 3 (9 months).	Keyes-Welch (110), Kallail (46), Kallail (73)
Trover campus rural pathways program, University of Louisville School of Medicine, Madisonville, USA	1998, 4-year graduate entry MD.	To provide two years of rural clinical training and reduce urban disruption in the training pipeline.	Western Kentucky (state). Distance from main campus (150 miles), population 20,000.	Rural scholars program for high school and college students.	Urban pre-clinical training followed by 2 rural clinical school years.	Keyes-Welch et al. (110), Crump et al. (39)
James Cook University (JCU), Queensland, Australia	1999, 6-year undergraduate bachelor’s degree.	To produce graduates who understand rural/remote, indigenous and tropical health issues, with the expectation that many will ultimately serve in those contexts.	Northern areas of Queensland (state). Population density 2.8/km^2^	High school outreach program, selection system aims to favour applicants from local rural towns.	Fully rural program, main campuses in rural locations.	Hays et al. (109), Hays et al. (114), Tesson (66), Veitch (143), Sen Gupta et al. (145), Larkins et al. (144), Sen Gupta et al. (124), Sen Gupta et al. (95), Schauer et al. (81), Woolley et al. (132), Larkins et al. (100), Woolley et al. (126), Woolley et al. (84), Reeve et al. (76), Woolley et al. (166), Larkins et al. (141), Ray et al. (123), Rourke et al. (58, 99, 106), Sen Gupta et al. (149), Woolley et al. (117, 119), Woolley et al. (78, 122, 127, 128), Woolley et al. (78, 122, 127, 128), Woolley et al. (78, 122, 127, 128), Woolley et al. (78, 122, 127, 128), Johnston et al. (75), Woolley et al. (49), Woolley et al. (116, 131)
University of California (UC) Davis Programs in Medical Education (PRIME), California, USA	2003, 4-year graduate entry MD.	UC Davis PRIME adopted a focus on underserved rural populations.	Rural areas of Northern California from Sacramento to the Oregon and Nevada borders (region).	Rural outreach programs. Multi-faceted admission assesses rural background, rural exposure, primary care practice interest.	Short rural placements throughout 4 years of the course, deliberately in different communities, rural clinical clerkships in year 3 (total 5 months).	Eidson-Ton et al. (70)
Northern Medical Program, University of British Columbia (UBC), British Columbia, Canada	2004, 4-year graduate entry program.	To admit and train future doctors who are more likely to locate their clinical practice in northern and rural settings.	Northern and rural areas of British Columbia (region). Size of Northern British Columbia 500,000/km^2^, population 300,000.	Remote suitability score used to assess suitable applicants for northern program.	4 months at urban campus then rest of program between 2 rural campuses and on clinical placements in Northern BC including rural and remote.	Bates et al. (98), Tesson et al. (66), Rourke (22), Snadden et al. (69), Snadden et al. (50)
One District One Doctor (ODOD) program, Ministry of Health, Thailand	2005, 6- year undergraduate.	National initiative to increase the production of doctors for rural and remote areas.	Rural districts.	Students are recruited from high schools in remote rural areas/non-provincial cities.	Urban pre-clinical training followed by 3 years of clinical training outside major cities and near their hometowns.	Putthasri et al. (107), Thammatacharee et al. (121), Nimkuntod et al. (120), Arora et al. (92), Techakehakij et al. (130), Somporn (60, 182), Techakehakij et al. (96), Figueiredo et al. (5)
Northern Ontario School of Medicine (NOSM), Northern Ontario, Canada	2005, 4-year graduate entry program.	To serve the healthcare needs of the people of Northern Ontario.	Northern Ontario (region). Statistics Canada Subdivisions: <10,000 people = rural. Population density 4/km^2^.	Preferences applicants from northern, rural, remote, Aboriginal or Francophone backgrounds.	Year 1 and 2 rural and Indigenous community placements (total 16 weeks), comprehensive community clerkship in year 3 (9 months).	Tesson et al. (66), Rourke (150), Rourke (22), Curran et al. (111), Strasser S et al. (153), Strasser R et al. (88), Strasser R et al. (112), Strasser R et al. (113), Strasser R et al. (113), Couper et al. (63), Strasser R et al. (151), Hudson (83), Hogenbirk et al. (129), Strasser R et al. (64), Hogenbirk et al. (80), Mian et al. (65), Reeve et al. (76), Larkins et al. (141), Mian et al. (139), Johnston et al. (75), Ross et al. (29), Woolley et al. (49), Grierson et al. (159), Hogenbirk et al. (79), Wood et al. (163)
Washington, Alaska, Montana, Idaho (WWAMI) TRUST program, University of Washington School of Medicine, Washington, USA	2008, 4-year graduate entry MD.	To redress physician maldistribution by increasing the number of students from the states of Washington, Alaska, Montana and Idaho being trained in primary care.	Rural areas of Washington, Alaska, Montana and Idaho states.	Preferential admission of students from rural areas or with substantial rural experience.	Students are connected to a single rural continuity site during 18 month pre-clinical curriculum, 1 month rural underserved opportunities program between 1^st^ and 2^nd^ year, 18-24 week longitudinal clinical rotation in rural continuity community.	Yergan et al. (140), Keyes-Welch et al. (110), Geyman et al. (77), Rabinowitz et al. (156), Schwarz (155), Hsueh et al. (87), Tesson et al. (66), Rourke (22), Kardonsky et al. (102)
Oklahoma State University Center for Health Sciences College of Osteopathic Medicine (OSU-COM), Oklahoma, USA	2011, 4-year graduate entry, DO.	To produce primary care physicians for rural practice with the skills to be effective community leaders and rural health advocates.	Rural areas of Oklahoma (state).	Early recruitment of rural high school students.	3-week rural experience between first and second year, years 3 and 4: required and elective rural-based clinical rotations with opportunity to complete most rotations rurally.	Keyes-Welch et al. (110), Wheeler et al. (57)
Northern Territory Medical Program (NTMP), Flinders University/Australia	2011, 4-year graduate entry or 6-year undergraduate entry MD.	To develop a medical workforce for the challenging Northern Territory environment.	Northern Territory. Whole of territory classified as rural, area 1.3 mill/km^2^, population 200,000.	Tiered admission system prioritises Indigenous and Northern Territory residents.	Fully rural-based program, main campuses in rural locations.	McDonnel Smedts et al. (42), Worley et al. (28)
Wisconsin Academy for Rural Medicine (WARM), The University of Wisconsin School of Medicine and Public Health, Wisconsin, USA	2015, 4-year graduate entry MD.	To increase the physician workforce by recruiting medical students who intend to practice in rural Wisconsin.	Rural areas of Wisconsin (state). 25% of state’s population live in rural areas.	Selected based on backgrounds, aptitude for and commitment to careers that focus on serving Wisconsin’s underserved rural populations.	Training based at rural campus in year 3.	Landeck et al. (162), Golden (161)
Uni of Kentucky College of Medicine (UK COM), Kentucky, USA	2015, 4-year graduate entry MD.	To address state physician shortages through the establishment of two 4- year regional campuses.	Rural counties in Kentucky (state).	Preferential admission of in-state applicants (goal 90%).	Fully rural program at 2 rural campuses.	Geyman et al. (77), Griffith et al. (90)
Inclusive track, Ministry of Health, Thailand	2017, 6-year undergraduate.	National initiative to increase the production of doctors for rural and remote areas (adaptation of CPIRD/ODOD program).	District/province.	Students are recruited from high schools in local remote rural areas.	Urban centre pre-clinical training followed by 3 clinical years located outside major cities and nearby their hometowns.	Techakehakij et al. (96)
West Virginia University (WVU) School of Medicine West Virginia, USA	Commencement date not available, 4-year graduate entry MD.	To provide primary care physicians who are trained to meet the medical needs of rural Appalachia.	Appalachian counties, including all of West Virginia (state).	Recruits rural students from rural Appalachian counties.	4-week rural rotation in third year in family medicine and 4-week community/rural rotation in 4th year.	Keyes-Welch et al. (110), Hedrick J (91)
University of Namibia SOM (UNAMSoM), Namibia	Commencement date not available.	To capacitate the health care workforce in Sub-Saharan Africa.	Unspecified geographical sub-regions of the country.	Regional quota system.	Rural experience year 1: 6 days, year 2: 3 weeks, year 3: once weekly for 36 weeks, year 5: extended placement (6 months).	Pasricha et al. (148), Eichbaum et al. (147)
University of New Mexico School of Medicine (UNM-SOM), New Mexico, USA	Commencement date not available, 4-year graduate entry MD.	Commitment to underserved urban and rural populations of New Mexico.	Rural areas of New Mexico (state).	Strong preference to recruit residents of New Mexico, minority groups and commitment to serve underserved groups.	9-week rural practical immersion in 1st year and clinical rotations and electives in rural areas in later years.	Geyman et al. (77), Tesson et al. (66)

**Table 3:** Characteristics of included non-medical programs, by year of commencement.

Program name, location	Year of commencement, health profession; program description	Purpose	Place definition	Place-based selection methods	Place-based rural training	Evidence sources/citations
Alabama Junior College/School of Health-related professions linkage, The University of Alabama Birmingham, Alabama, USA	1971, Allied Health; undergraduate.	Provide integrated Allied Health Professional training that is accessible to those living in rural areas of the state, to improve workforce outcomes.	Rural areas of Alabama (state).	Recruitment partnership with junior colleges	First year at urban campus followed by full time clinical placement often in home rural area	Joiner 1992 (68)
The Area Health Education Centre (AHEC), multi-agency consortia in 20 southern counties, Texas, USA	1973, Allied Health; undergraduate.	To improve health manpower needs by decentralizing training for allied health and recruiting local students and training them in local areas.	20 southernmost counties of Texas (region).	Recruitment of students from local area.	Trains students at local sites.	Philips 1978 (43)
University of Wisconsin-Eau Claire program, University of Wisconsin, Wisconsin, USA	1986, Nursing; undergraduate.	To prepare more baccalaureate prepared nurses for rural areas of central Wisconsin and to accommodate the educational needs of geographically bound non-traditional students.	Rural areas of central Wisconsin.	Designed to be accessible to geographically bound students.	Blended learning/distance education delivered to students at an off campus rural site.	Ostmoe 1989 (55)
Tromso Distance Nursing Education (DNE), Tromso University College, Tromso, Norway	1990, Nursing; 4-year part-time undergraduate.	To offer higher education to students living in rural communities and strengthen the workforce in selected municipalities.	Northern Norway, rural region of Tromso.	Students document connection to one of the program’s 18 rural municipalities.	Blended learning/distance education delivered to students in Troms region of Northern Norway.	Norbye 2013 (85)
The Arctic University of Norway-Hammerfest, County, Norway	1991, Nursing; 3-year undergraduate.	To recruit and educate nurses for Finnmark county	Finnmark County, population 75,860, area 48,631/km^2^, 18 of 19 local authority areas defined as rural with fewer than 2 people/km^2^.	Local recruitment: the location of the study sites determines where the students come from.	Blended learning/ distance education delivered in 9 rural communities in Finnmark.	Eriksen 2019 (86)
Ara Institute of Canterbury, Otautahi (Christchurch), Aotearoa (New Zealand)	2000, Bachelor of Midwifery; 4-year undergraduate.	To enable students to access education from their own communities, to address midwifery workforce shortages in rural and provincial areas of the upper Te Waipounamu.	Distributed across the Upper Te Waipounamu, with satellite sites across Canterbury, as well as Nelson, Marlborough and the West Coast.	Students are recruited from across the upper half of Te Waipounamu (South Island).	Blended learning/distance education enabling students to complete most of the program in the areas in which they live.	Daellenbach 2022 (82)
Pipeline, Profession and Practice (PPP): Community-based Dental Education Program, Ohio State University College of Dentistry (COD), Ohio, USA	2002, Dentistry; Graduate entry.	To increase access to dental care for underserved populations.	Rural areas of Ohio (state).	75% of places are reserved for students from Ohio. Active recruitment to increase diversity of cohort.	Short rural rotations during fourth year.	Thind 2009 (59)
Charles Sturt University (CSU), New South Wales, Australia	2003, Bachelor of Nursing; Undergraduate.	To enable enrolled nurses to upgrade to registered nurses without leaving their communities.	New South Wales and Northern Victoria (region).	Community outreach program to recruit potential applicants.	Blended learning/distance education delivered to students living in target communities.	Latham et al. 2009 (41)
Web-based Bachelor of Science in Pharmacy program, Umea University, Sweden	2003, Pharmacy; 3-year undergraduate.	To address the shortage of prescriptionists in rural Northern Sweden.	Focus on 3 rural counties in Northern Sweden.	5 local study sites located in strategic rural areas to attract local students.	Blended learning/distance education. Local study groups facilitate the recruitment of students living in target areas and have them remain there.	Mattsson et al. 2018 (47)
The Pathway Program (PP), California social work education network, California State University, California, USA	2006, Social work; undergraduate.	To develop a distance education program for employees of county and tribal health and human services departments in rural California.	Aims to serve ‘struggling California’ – isolated regions of Northern California and Inland southern California (region).	Initially aimed to recruit employees of child welfare agencies, extended over time to other under-represented students.	Blended learning/distance education delivered to students living in target communities.	Morris et al. 2016 (38)
Missouri Health Professions Consortium Occupational Therapy Assistant Program (MHPC-OTA), a consortium of 5 community colleges in Missouri, USA	2008, Occupational Therapy Assistants; undergraduate.	To provide educational opportunities to rural, place -bound students.	Rural areas of Missouri (state).	Recruitment targets students currently embedded in rural communities.	Blended learning/distance education through one of 5 community colleges covering most areas of the state.	Brandt 2014 (54)
Rural Pharmacy Education (RPHARM) program, The University of Illinois at Chicago College of Pharmacy, Rockford, Illinois, USA	2010, Pharmacy: an interprofessional rural health professions program (linked with Illinois RMED).	To graduate pharmacy and medical students who will return to live and work in rural Illinois.	Rural areas of Illinois (state). Rockford campus 90 miles from Chicago.	Targeted recruitment in rural communities classified as Rural Urban Commuting Area (RUCA) 4 or higher, multi-faceted admission seeking rural background, rural interest, community recommendation.	Short clinical placements in rural communities (3 × 6 weeks).	Soliman et al. 2012 (97)
Plan of Dentistry (PoD) North Carolina. School of Dentistry at East Carolina University and University of North Carolina Chapel Hill, North Carolina, USA	2011, Dentistry; 4-year.	Educating primary care dentists for rural and underserved areas of North Carolina.	Rural areas of North Carolina (State). Non-metropolitan counties considered rural areas.	Students recruited based on mission of the school and community service. Collaboration plans with community colleges.	Fourth year program in 8–10 service-learning centers in underserved rural communities.	Chadwick et al. 2008 (67)
The College of Nursing, University of Saskatchewan, Northern Saskatchewan, Canada	Commencement date not available, nursing; undergraduate.	Widening access for students in Northern Indigenous communities to study nursing.	Northern Saskatchewan province. Population of 38,000 people across 45 communities.	Focus on recruiting Indigenous residents from Northern Saskatchewan province.	Fully rural program at three distributed rural sites.	Butler et al. 2018 (37)
Eastern Shore of Virginia (ESVA) RN-to-BSN, Virginia, USA	Commencement date not available, nursing; undergraduate.	To increase the number and diversity of baccalaureate educated nurses from a rural resource-limited community in the United States.	Rural Eastern Shore of Virginia.	Recruitment of students from the economically disadvantaged rural area.	Blended learning/ distance education delivered to students living in target community.	Hawkins et al. 2018 (89)
Registered Nurse/Bachelor of Science in Nursing Public Health Leadership in Nursing Program, he University of Wyoming and Health Resources and Services Administration’s (HRSA) Nurse Education, Practice, and Retention Grant Program, Wyoming, USA	Commencement date not available, nursing; undergraduate.	RN to BSN completion program designed to develop strong public health leaders for underserved rural communities.	Rural communities of Wyoming (state). State population density of 5.1 people per square mile and 73.9% of the population living in frontier areas of the state.	Targeted recruitment of students in community colleges and the community, with a focus on underrepresented groups.	Blended learning/distance education delivered to target populations of students in rural underserved communities.	Ouzts et al. 2006 (52)
Rural Clinics Program, University of Texas Arlington, Texas, USA	Commencement date not available, nursing; undergraduate.	Preparing nurses for rural Texas.	Rural Texas (state).	Program delivered to existing residents of rural communities.	Education delivered by visiting faculty to students who remain living in their rural communities.	Sandlin 1994 (115)
Delta Health Education Partnership (DHEP), partnership of six schools spanning four states – Arkansas, Louisiana, Mississippi and Tennessee, USA	Commencement date not available, nursing & midwifery.	Recruit, educate and retain interdisciplinary groups of primary healthcare practitioners to increase access to healthcare.	Areas of the lower Mississippi Delta that are medically underserved and experience health professional shortages.	Active recruitment of students from non-traditional backgrounds and minority groups and with a commitment to practice in the region as graduates.	Blended learning/distance education delivered to students living in target communities.	Skorga 2002 (53)

#### Conceptualisation and purpose

The concept of a ‘place-based’ approach was evident in terminology used by many programs, although only one used this precise term (29). Concepts used to describe place-based programs included ‘learn where you live’ (37) and ‘grow your own’ (28, 38). Other place-related language included ‘sense of place’ (acknowledging the existence of place connection, importance of access and opportunity and approaches to strengthening place connection through training) (37, 39), ‘local’ [usually with reference to the location of the medical school) (40–44), ‘home’ or ‘hometown’ (in relation to allowing students to study in or near their home communities (38, 45–47) or evaluating the retention of students or graduates in their home areas (47–51)] and ‘place-bound’ referring to rural nursing or allied health students with reduced geographic mobility restricting educational access (52–55).

All 50 programs had a clear purpose related to improving rural workforce outcomes. Programs with a predominant workforce purpose used terminology such as ‘rural pipeline’ (20, 21, 39, 44, 56–60) or ‘rural track’ (57, 61, 62) to describe a deliberate approach to provide a separate stream of recruitment and training specifically focussed on achieving rural workforce outcomes. Programs were also described as being ‘comprehensive’ (20, 21, 57, 63, 64) when they included multifaceted, coordinated initiatives including targeted recruitment, admissions, curriculum, support, and evaluation components spanning the training continuum to achieve workforce goals.

There was an equity-driven purpose identified in many programs, underpinned by a social accountability framework. This was evident through the frequent use of the term ‘underserved areas’ (5, 39, 43, 53, 54, 56, 59, 65–72) to describe priority areas for workforce improvements. The term ‘underserved’ was often not precisely defined but was frequently inclusive of rural areas (rural underserved) but also extended the program’s focus beyond rural areas alone (rural and underserved). Underserved areas were sometimes defined by medical programs as having low physician to population ratios (61, 73, 74).

The majority of programs (*n* = 38/50) demonstrated a social accountability mandate through a concurrent purpose to address both rural workforce needs and widen access to their educational programs for students from rural areas or other under-represented populations. Population groups that were targeted under widening access measures related to each program’s context, and included Indigenous populations (Australia, Canada, USA), Francophone speakers (Canada), and those experiencing socio-economic or socio-cultural disadvantage (USA) (75). A dual focus on widening access and workforce outcomes was more prevalent in the non-medical programs.

Social accountability was also evident in terminology applied to community-centered educational models, including community-‘engaged’ (29, 37, 76), ‘oriented’ (77) or ‘based’ (76–78). Educational models that emphasized rural community-based learning to increase access for rural students were described as ‘distributed’ (37, 58, 79–83), ‘decentralised’ (43, 58, 77, 78, 81, 84–87), ‘distance education’ (37, 38, 41, 47, 53, 54, 67, 88, 89),’ learn where you live’ (37) or as a ‘flipped model’ (49).

### Research question 2: how is this concept translated into pre-registration HPE program design?

#### How is ‘place’ defined geographically?

Often very large jurisdictional sub-divisions, such as states, territories, and provinces were used by programs in the USA (*n* = 12), Canada (*n* = 1), and Australia (*n* = 2) to define a program’s place of focus. This approach was particularly evident in the USA where the workforce purpose of programs was frequently stated in terms of providing a workforce for rural areas of the state in which the program was based, for example, ‘training Kentuckians in Kentucky to practice in Kentucky’ (90). Reference to one or more rural counties was also used to further clarify geographic focus, evident in the USA (43, 90, 91) and European programs (47, 86). For example, Umea University’s web-based pharmacy program focussed on three rural counties in Northern Sweden in which local study groups were established (47). Countries smaller in land mass tended to use smaller geographic areas to define their place-based focus, assisted by higher resolution national-level geographic subdivisions such as provinces in Thailand (92), and prefectures in the Philippines (93) and Japan (94).

Approximately one-third of programs (*n* = 16) employed descriptive approaches alone to define their region of focus, specific to their context and with an absence of reference to any political or geographical rural subdivisions. Examples include ‘northern’ (37, 38, 95) ‘western’ (39) or ‘southern’ (41, 43) areas of a state or province, or the description of geographical landmarks or boundaries defining an area (53, 70, 82). For example, the University of California Davis Programs in Medical Education (PRIME) describes their region as ‘rural Northern California regions from Sacramento to the Oregon and Nevada borders’ (70).

The rurality of program locations was inconsistently defined, often without reference to a formal geographic classification system. When provided, descriptors included the proportion of the region defined as rural by statistical measures, population density, population and/or size of the region or training site, distance of training sites from major campuses or cities, and physician-to-population ratios (Tables 2, 3).

#### How is place-based student recruitment occurring?

Although all programs included some form of rural student recruitment, detail was often lacking to determine precisely how this was occurring, and the extent to which this was place-based. At a strategic level, the establishment of rural campuses in areas of workforce shortages was a mechanism used to attract local applicants to programs (39, 69, 90). It was observed that the location of rural learning sites for distance education programs heavily influences who participates in these programs (20, 22, 47, 70).

Recruitment in many cases began some years ahead of health professional training commencement, through the provision of formal pre-entry pathways (39, 44, 46, 56). Common in the USA, pre-entry pathways often included cooperative arrangements with undergraduate institutions in the state (39, 74), and offered guaranteed admission if academic standards were maintained (44, 46, 56). Further targeted pre-entry recruitment strategies were implemented by programs within their communities, including quota-based arrangements with local high schools (92, 94, 96) and active marketing and recruitment (41, 52, 53, 59, 67, 97). Pre-entry programs provided students with clear entry pathways to health professional training as well as often providing opportunities for rural and healthcare exposure to consolidate rural interest prior to course commencement (39, 56).

At the point of course entry, a wide range of approaches were used that attempted to identify and select students aligned with the goals of the program. There were only two examples of admission processes that aimed to identify suitability for a specific place: the Remote Suitability Score developed by the University of British Columbia for their Northern program (98), and Northern Ontario School of Medicine’s (NOSM) context score, aligned with the admission of students reflecting Northern Ontario’s population demographics (22). Other programs reflected on the influence of the learning locations on the students who participated, leading to the conclusion that study sites can be strategically located to recruit students from target areas of workforce shortage (47, 86).

Admission quotas were used on a national (94) or regional scale (40, 42, 46, 75, 87, 99, 100) to facilitate preferential admission for rural students. Preferential admission of students from the area of focus (28, 100, 101) or rural areas generally (21, 44, 102–104) was also mentioned without stating a specific quota requirement. Demonstration of rural residence or background in the region of focus was commonly a requirement, although detail was not always provided on how this was assessed. Mechanisms that were described included providing evidence of a minimum number of years spent living in a defined rural region (44, 62, 74), small hometown (21, 44, 104–106), or attending a rural high school (44, 46).

Tailored admission requirements were commonly observed across program types, including a reduced emphasis on academic attainment (39, 40, 44, 100), program-specific exams (40, 107), waiver of aptitude tests (39, 108), and score adjustments for educational disadvantage (99, 109). Some medical programs retained the full admission requirements of the main medical program with additional requirements for the rural pathway, such as current or previous residence in a rural underserved area (74, 102), interviews (20, 70, 101, 102), and letters of recommendation (20, 74).

Several studies described mechanisms for community involvement in student recruitment, with their contributions including personal letters of recommendation (20, 74, 97), student nomination/endorsement (62, 103), involvement in interviews (100), and admission committees (20, 44).

Beyond seeking students from a rural background, admissions processes were often employed to identify students considered more likely to enter rural and/or primary care practice. Assessment of rural identity and intent for rural practice was sought through personal statements (22, 98, 109) and admissions committee interviews (20, 44, 46, 56, 59, 70, 74, 99, 100, 102, 109), by seeking pertinent life experiences and characteristics (104), and rural lifestyle/hobbies (44, 98). Several USA programs had a strong focus on recruiting students with an intention for family medicine practice (20, 46, 74, 110) or generalist practice (44, 70). Evidence of community service was sought as a characteristic of students suitable for program participation (20–22, 59, 70, 104) and, in medicine, this was deemed to be a trait associated with future family medicine practice (21, 77).

#### How are place-based training and recruitment co-occurring?

The authors of this review have classified a typology of included programs according to four main models of rural training delivery (Table 4).

**Table 4:** Typology of rural training models by health profession.

	Type 1: short rural placements	Type 2: extended rural placements	Type 3: rural campuses	Type 4: distributed blended learning	Not able to be classified
Description	*Urban primary campus location with recurrent short rural clinical placements (<12 weeks)*	*Blend of urban campus training with extended rural placements (>12 weeks), including Longitudinal Integrated Clerkships*	*Fully rural program, including pre-clinical campuses*	*Fully distributed rural program, entire program delivered to students in rural locations through blended learning/distance education.*	*Rural training model not specified*
Medicine	Regional quota scheme Jichi Medical University and Asahikawa Medical University, Jefferson Medical College Physician Shortage Area Program, West Virginia University School of Medicine, University of New Mexico School of Medicine, University of California Davis Programs in Medical Education	Thailand Ministry of Health (MOH) Collaborative Project to Increase Production of Rural Doctors, Thailand MOH One District One Doctor, Thailand MOH Inclusive Track, University of Missouri School of Medicine Rural Track Pipeline Program, Michigan State University Upper Peninsula Program, University of Louisville School of Medicine Rural Pathways Program, University of Minnesota Medical School Rural Physician Associate Program, University of Illinois College of Medicine Rural Medical Education track, University of Alabama Rural Medical Scholars Program, University of Washington School of Medicine Washington, Alaska, Montana, Idaho TRUST program, The University of Wisconsin School of Medicine and Public Health Wisconsin Academy for Rural Medicine, University of Kansas School of Medicine Scholars in Primary Care Program, Oklahoma State University Center for Health Sciences College of Osteopathic Medicine, Ateneo de Zamboanga University School of Medicine, University of Philippines School of Health Sciences, Chulalongkorn University Medical Education for Students from Rural Areas Project, University of Namibia School of Medicine, Walter Sisulu Medical School, Northern Ontario School of Medicine, University of British Columbia Northern Medical Program, Memorial University of Newfoundland and Labrador	James Cook University, Northern Territory Medical Program, University of Kentucky College of Medicine, West Virginia School of Osteopathic Medicine		University of Gezira Faculty of Medicine, University of Tromso Medical School
Nursing				The College of Nursing, University of Saskatchewan, Charles Sturt University, Ara Institute of Canterbury, The Arctic University of Norway-Hammerfest, Tromso University College Distance Nursing Education, Eastern Shore of Virginia Registered Nurse-to-Bachelor of Science in Nursing program, University of Wisconsin-Eau Claire program, University of Wyoming and Health Resources and Services Administration’s Nurse Education, Practice, and Retention Grant Program, University of Texas Rural Clinics Program, Delta Health Education Partnership	
Dentistry	Ohio State University College of Dentistry Pipeline, Profession and Practice program	School of Dentistry at East Carolina University and University of North Carolina Plan of Dentistry			
Allied Health		The University of Alabama Linkages program			Area Health Education Center, Texas
Pharmacy	The University of Illinois at Chicago College of Pharmacy Rural Pharmacy Education program			Umea University Web-based Bachelor of Science in Pharmacy program	
Social work				California State University social work education network Pathway Program	
Occupational Therapy				Missouri Health Professions Consortium Occupational Therapy Assistant Program	

In *Type 1 programs: short rural placements* (medicine: *n* = 5, dentistry: *n* = 1, pharmacy: *n* = 1), rural stream students are attached to a larger mainstream urban program and participate in one or more short term rural rotations. The duration varies from 8 weeks of clinical placement to multiple separate short-term rotations across all years of the course. For example, at the University of New Mexico (USA), students complete a practical rural immersion in their first year, and clinical rotations in rural areas in later years (66). Although programs in this category offer training within the program’s defined rural region of varying durations, it was often not clear whether mechanisms were in place to allow students to experience continuity with a single rural community, or their home community, when they undertake more than one rural placement during their course. Jichi Medical University (Japan) reported that students spent ‘some weeks’ in their home prefectures in 5th year (94). In contrast, UC Davis rural-PRIME (USA) rotates students between five different rural communities for their third-year clerkships to experience different systems of care.

In *Type 2 programs: extended rural placements* (medicine: *n* = 21, dentistry: *n* = 1, allied health: *n* = 1), rural stream students complete pre-clinical training at an urban campus but undertake at least one extended period of rural placement (≥12 weeks). This model describes most included medical programs, although there is considerable variation in the structure and timing of rural placements. Several programs are structured around a number of years of initial urban campus-based pre-clinical training followed by relocation for subsequent years of rural clinical training. An example is the Collaborative Project to Increase Production of Rural Doctors program (Thailand) where rural track students complete their first three years jointly at universities with ‘normal track’ students, then relocate to regional and provincial hospitals in their home communities for their three clinical years (92, 107). In other programs, such as NOSM (Canada), students participate in several eight-week rural immersion experiences in the early years, followed by an eight-month community-based rural longitudinal integrated clerkship in third year (111, 112).

There were few examples of programs of this type that deliberately connect students with a single rural community or their home region for rural placements (68, 99, 102). An exemplar is the Targeted Rural Underserved Track (TRUST, USA), which provides students with a longitudinal curriculum connected to a single rural continuity site, throughout the course duration. By contrast, NOSM deliberately provides students with a diverse range of rural community placements in Northern Ontario (113). Most other programs of this type did not provide information on whether students’ rural placements provided continuity with their home or another rural community over time.

In *Type 3 programs: rural campuses* (medicine: *n* = 4), the entire training duration occurs in a rural location. These programs were confined to medical schools established in rural states or areas. Australian examples are the Northern Territory Medical program and James Cook University, with all four years of delivery based at rural campuses and training sites. Preferential student recruitment to these programs occurs at the level of the state or region of the medical school, suggesting that students may be required to relocate from their home communities to attend these rural campuses. The included articles did not provide details on the process by which students were matched to a rural training campus to determine whether their home rural community was a key consideration. An expectation of these programs was that the establishment of a new rural campus would attract students from that location into the course, who would then choose to train there (69, 114).

In *Type 4 programs: distributed blended learning* (nursing & midwifery: *n* = 9, occupational therapy: *n* = 1, pharmacy: *n* = 1), confined to nursing & midwifery and allied health programs, students complete their studies without leaving the rural communities in which they live (37, 38, 41, 47, 53, 54, 67, 88, 89). Learners are often more widely dispersed across a rural region and in smaller groups, with education delivered through blended learning, combining online curriculum delivery with local, site-based teaching and supervision. Decentralized training sites were often strategically established in areas of workforce shortages, with small groups of students working with local clinical preceptors (52, 89, 115). An example of this model is the Eastern Shore of Virginia Registered Nurse to Bachelor of Science in Nursing program delivered via online asynchronous distance learning, in partnership with local hospital preceptors and study facilities.

#### What program outcomes are being investigated?

The scope of outcomes evaluated was broad, with three main areas studied: (i) graduate workforce outcomes, (ii) success in widening access to HPE programs, and (iii) academic outcomes. In addition, a range of other impacts of programs were evaluated, with a full list available in Table 5.

**Table 5:** Outcomes evaluated by programs.

	**Outcome measure**	**Programs**
**Workforce outcomes**		
*Graduates’ Geographic Work Locations*	Working rurally: non-Standard Metropolitan Statistical Area (SMSA) counties (USA)	Medicine: University of Minnesota Medical School Rural Physician Associate Program (RPAP) (157), University of Missouri School of Medicine Rural Track Pipeline Program (MU-RTPP) (56), Jefferson Medical College Physician Shortage Area Program (PSAP) (39, 74, 110, 157), West Virginia University School of Medicine (WVU) (104), University of Washington School of Medicine Washington, Alaska, Montana, Idaho TRUST program (WWAMI) (77, 110, 157) Allied Health: The University of Alabama Linkages program (UAL) (68, 115)
	Working rurally: RUCA codes 4-10 (USA)	Medicine: RPAP (135), University of Illinois College of Medicine Rural Medical Education track (RMED) (20)
	Working rurally: Statistics Canada census subdivisions < 10,000 (Canada)	Medicine: Northern Ontario Medical School (NOSM) (79, 129), University of British Columbia Northern Medical Program (UBC) (58, 133, 134), Memorial University of Newfoundland and Labrador (MUN) (58, 134)
	Working in rural community <10,000, rural town <30,000	Medicine: MUN (106)
	Working rurally: Australian Standard Geographical Classification System Remoteness Areas (ASGS-RA) 2-5 (Australia)	Medicine: James Cook University (JCU) (124, 132, 167)
	Working rurally: Modified Monash Model (MM) 2-7 (Australia)	Medicine: JCU (131)
	Working in rural towns <25000, <30000 or <50000	Medicine: RPAP (19, 157), Michigan State University Upper Peninsula Program (UPP) (56, 101, 157), PSAP (157), Regional quota scheme Jichi Medical University (JMU) (166), UBC (106), JCU (116)
	Working rurally: remote village, small town, large town	School of Health Sciences, University of Philippines (UPM-SHS) (103)
	Working rurally: not defined	Medicine: (UPP (155), PSAP (5, 77, 161), West Virginia University School of Medicine (WVSOM) (104), University of Alabama Rural Medical Scholars Program (RMSP) (44), RMED (19), NOSM (152), UBC (22, 64), JCU (143, 144), Ateneo de Zamboanga University School of Medicine (ADZU-SOM) (93, 144), Chulalongkorn University Medical Education for Students from Rural Areas Project (MESRAP) (146), Walter Sisulu Medical School (WSU) (58, 159) Occupational therapy: Missouri Health Professions Consortium Occupational Therapy Assistant Program (MHPC-OTA) (54),
	Working in state, territory, province or prefecture or other described region of medical school	Medicine: RPAP (5), MU-RTPP (56), RMSP (44) RMED (19) WWAMI (77, 110), Northern Territory Medical Program (NTMP) (28, 42), UPP (155), University of Tromso Medical School (Tromso) (48), NOSM (49, 64, 79, 129, 152), JCU (49, 128, 131, 143), ADZU-SOM (93, 113) Occupational Therapy: MHPC-OTA (54) Pharmacy: Umea University Web-based Bachelor of Science in Pharmacy program (Umea)(47)
	Working in region of clinical training	Medicine: JCU (81, 84), WSU (159) Allied Health: UAL (68)
	Working in home state, territory, province or prefecture of origin	Medicine: WWAMI (77, 156), JMU (94, 166), Tromso (48), UBC (64, 106, 134)
	Working in hometown or home area of origin	Medicine: JCU (81) Nursing: Ara Institute of Canterbury (Ara) (82), AUNH (86) Allied Health: UAL (68, 115), AHEC (43),
	Working in underserved areas/intention to do so	Medicine: PSAP (5, 110) PSAP (74, 77, 110)/JCU (141), WSU (141), Gezira (141), ADZU-SOM (93, 100, 119, 141), UPM-SHS (103)
	Motivation or intention to relocate or emigrate	Medicine: WVU (91), JMU (165), Tromso (48), NOSM (75), JCU (75), WSU(75), Gezira (75), ADZU-SOM (75), UPM-SHS (75).
	*Graduate career time points*	Intentions at entry and exit from medical school	Medicine: NOSM (75), JCU (75, 116, 124–126, 128, 141), WSU(75, 141), Gezira (75, 141), ADZU-SOM (75, 141), UPM-SHS (75), JCU (143) Dentistry: PPP (59)
Internship or first practice		Medicine: RPAP (135), MU-RTPP (56), JCU (58, 81, 84, 124, 126, 128, 143, 144), UPM-SHS (103)Nursing: The Arctic University of Norway-Hammerfest (AUNH) (86), Tromso University College Distance Nursing Education (DNE) (85) Allied Health: AHEC (43) Occupational Therapy: MHPC-OTA (54)
Early career (1-5 years after graduation)		Medicine: RMED (19, 61), RMSP (44), UPP (101), NOSM (79, 152), UBC (106, 133), JCU (95, 122, 144, 167), ADZU-SOM (93), WSU (58), Pharmacy: Umea (47) Allied Health: AHEC (43),
End of mandatory training or return of service		Medicine: JMU (5, 45, 71, 72, 166), NTMP (28)
Mid-career (5-10 years after graduation)		Medicine: PSAP (74, 137, 157), WVSOM (104), Tromso (48), NOSM (79, 129), UBC (134), JCU (49, 95, 117, 131, 132, 167), ADZU-SOM (93, 118, 119), UPM-SHS (103, 119) Nursing: AUNH (86), DNE (85) Pharmacy: Umea (47)
Late career (>10 years after graduation)		Medicine: PSAP (5, 19, 161), WWAMI (77), UBC (134), UPM-SHS (103), RPAP (19) WVSOM (77, 104), UBC (134), JCU (131), UPM-SHS (103, 119), RMED (20)
Long term retention in rural practice		Medicine: PSAP (5, 19, 138, 161), WWAMI (77)
*Specialty/setting*	Completion of Family Medicine/General Practice training/residency	Medicine: PSAP (74, 138), RMSP (44, 142), NOSM (79, 152), UBC (106), JCU (49, 95, 144), UPM-SHS (103)
	Current fully qualified practice in Family medicine/General practice	Medicine: RPAP (135, 157, 158), UPP (87, 155, 157), MU-RTPP (56) PSAP (5, 74, 77, 161), RMED (20, 21), WWAMI (102), JCU (131)
	Family medicine, long-term retention	Medicine: RPAP (138, 161)
	Primary care (family physician, paediatrics or general internal medicine)	Medicine: RPAP (158), UPP (155), PSAP (77, 110, 157), RMSP (44), RMED (20, 21), WWAMI (77, 102, 110, 157), JMU (94), Tromso (48)
	Scope of practice, generalist practice	Medicine: Trover (110), NOSM (79), JCU (78, 116, 123), ADZU-SOM (118)
	Working in public/government/community health care	Medicine: JCU (117)ADZU-SOM (118), UPM-SHS (103) Nursing: DNE (85) Pharmacy: Umea (47)
	Working with underserved population groups/intention to	Medicine: NOSM (79, 160), JCU (100, 117, 122), ADZU-SOM (100, 119), UPM-SHS (76), WSU (100), UG (100) Dentistry: PPP (59)
*Workforce contribution*	Graduates as proportion of rural physicians in state/province	Medicine: PSAP (5, 74, 138), UBC (133)
	Availability of physicians in small communities or municipalities, reduction in recruitment expenditure and reliance on locum physicians.	Medicine: ADZU-SOM (76, 93), NOSM (65)
	Number of junior resident medical staff in the region	Medicine: JCU (144)
	Student roles in physicians’ offices	Medicine: RMED (21)
Widening access through student recruitment	Proportion of rural students: rural hometown or primary school	Medicine: RPAP (105, 135), RMED (21), NOSM (75, 79) UBC (22, 99, 106), JCU (75, 141, 144), WSU (75, 141), Gezira (75, 141), ADZU-SOM (75, 141), UPM-SHS (75). Pharmacy: RPHARM (97)
	Proportion of rural students: rural not defined	Medicine: UPP (39), Trover (39), JCU (114, 149)
	Proportion of students from state, province, prefecture, territory, county or other described region of school/program	Medicine: RMED (21), JCU (114, 149) NTMP (28), UPP (87), Trover (39), WVU (91), Tromso (48), NOSM (64, 88, 108, 152) Nursing: AUNH (86), DNE (85), Eastern Shore of Virginia Registered Nurse-to-Bachelor of Science in Nursing program (ESVA) (89) Pharmacy: Umea (47)
	Proportion of students with low socio-economic status	Medicine: JCU (75, 141), WSU (75, 141), Gezira (75, 141), ADZU-SOM (75, 118, 141), UPM-SHS (75), NOSM (75)
	Proportion of Aboriginal students	Medicine: NOSM (79, 88, 152), UBC (99), NTMP (28), JCU (144)Nursing: UoS (37)
	Proportion of Francophone students	Medicine: NOSM (79, 88, 152)
	Proportion of students who identify with underserved group	Medicine: JCU (75, 100, 141), WSU (75, 141), University of Gezira Faculty of Medicine (UG)(75, 141), ADZU-SOM (75, 141), NOSM (75), UPM-SHS (75) Dentistry: Ohio State University College of Dentistry Pipeline, Profession and Practice program (PPP) (59)
	Parental income, educational level	Medicine: JMU (94), JCU (141), WSU (141), UG (141), ADZU-SOM (141), UPM-SHS (75, 103)
Academic and competency-based outcomes	Comparison of rural and mainstream cohort grades	Medicine: PSAP (77), RMSP (44), RMED (21), NOSM (152), UBC (69), NTMP (28)
	Student attrition/retention	Medicine: MU-RTPP (56), WWAMI (140), University of California Davis Programs in Medical Education (PRIME) (70) Nursing: The College of Nursing, University of Saskatchewan (UoS) (37, 86), ESVA (89)
	Graduation from program	Medicine: NTMP (28)
	Achievement of clinical competence: procedural skills, biomedical and clinical knowledge, achievement of learning outcomes, patient/problem encounters, work readiness	Medicine: RPAP (158), RMED (21), NOSM (152), JCU (122), UPM-SHS (103), MESRAP (146)
	Social accountability competencies: communication skills, teamwork, professionalism, commitment to health equity and working with underserved populations	Medicine: JCU (76, 116, 122), ADZU-SOM (118), UPM-SHS (103), RMED (21) Occupational Therapy: MHPC-OTA (54)
	Progression to post-graduate degree and residency programs	Medicine: PSAP (161), RMED (21) Occupational Therapy: MHPC-OTA (54)
	Medical licencing or certification exam results	Medicine: RMSP (44), RMED (20), JMU (45), NOSM (152), ADZU-SOM (93, 113, 144), UPM-SHS (103) Occupational Therapy: MHPC-OTA (54)
Community outcomes	Correlation of community projects with community needs, impact of community projects	Medicine: RMED (21), ADZU-SOM (93)
	Community empowerment and economic impacts	Medicine: NOSM (152)
	Decrease in infant mortality rates	Medicine: ADZU-SOM (93, 144)
Economic outcomes	Contribution of physician tutors (economic and teaching)	Medicine: RPAP (158)
	Increase in graduates’ household income	Medicine: ADZU-SOM (118) Occupational Therapy: MHPC-OTA (54)
University outcomes	Graduates serving as faculty	Medicine: MU-RTPP (56)
	Reasons for applying to rural programs	Medicine: JMU (71), JCU (128), UPM-SHS (103) Nursing: DNE (85) Pharmacy: Umea (47)
	Barriers and facilitators to program entry	Medicine: WVU (91) Nursing: Charles Sturt University (CSU) (41) Allied health: Umea (47) Occupational Therapy: MHPC-OTA (54)
	Number of placement weeks spent in rural communities	Medicine: UBC (99)

Most program outcomes were evaluated through cross-sectional and retrospective single or multi-cohort studies using surveys (39, 43, 49, 78, 79, 81, 85, 97, 101–103, 107, 116–129), data linkage of school-held administrative data with publicly available practice location data (42, 43, 47, 56, 58, 74, 79, 84, 92, 95, 106, 130–138) or admissions data analysis (139, 140). Other types of studies included prospective cohort studies (54, 75, 100, 108, 141–143), case studies (44, 60, 62, 65, 91, 93, 99, 144, 145), program descriptions/project reports (20, 21, 28, 37, 38, 46, 50, 52, 53, 55, 57, 61, 68–70, 73, 83, 86, 88–90, 96, 98, 109, 112–115, 146–155), reviews (5, 19, 76, 77, 87, 156, 157), commentaries/policy analysis (22, 41, 113, 144, 158–160), editorials (64, 105, 161, 162), an economic review (80), a conceptual framework (163), and a typology (66).

Early program evaluations demonstrated an interest in the academic equivalence of rural stream students to their urban counterparts, through measures including assessment results (21, 28, 44, 69, 77, 151), clinical competency assessments (21, 103, 122, 146, 151, 157), development of social accountability competencies (54, 76, 103, 116, 118, 122), attrition and retention rates (37, 56, 70, 86, 89, 140), medical licensing exam results (44, 45, 93, 103, 113, 135, 144, 151) and progression to post-graduate degrees and residency programs (21, 54, 160). One program reported distributed learning as the most important factor in increasing the success of Indigenous students in nursing education (37).

Early graduate workforce outcomes (1–5 years) evaluated included graduates’ career intentions when entering and exiting medical school (75, 116, 124–126, 128, 143), first practice locations (43, 54, 56, 58, 81, 84–86, 103, 124, 126, 128, 135, 143, 144), and intention to emigrate (48, 75, 91, 164). Programs evaluating mid-career (5–10 year) graduate outcomes investigated the impact of specialty training vocation on rural practice (74, 79, 103, 118, 128, 129, 131, 134, 137), and retention in rural practice (74, 85, 156), including after the completion of mandatory training or return of service periods (5, 28, 45, 71, 72, 165). Longer-established programs evaluated outcomes of multiple cohorts, including rural practice locations in late career (>10 years) (5, 19, 20, 77, 103, 104, 119, 131, 134, 160), and long-term graduate retention in rural practice (5, 19, 77, 138, 160).

Locally relevant rurality classification systems were used to determine the number of graduates in rural practice (Table 5). In the USA, these included non-Metropolitan Statistical Area counties (39, 56, 68, 74, 77, 104, 110, 115, 156) and Rural–Urban Communing Area (RUCA) codes (20, 135). Canadian programs used Statistics Canada census subdivision of <10,000 population as the definition of a rural community (58, 79, 106, 129, 133). Australian programs utilized the Australian Statistical Geography Standard Remoteness Areas (ASGS-RA) (124, 132, 166) and Modified Monash Model (MM) (131) to classify the rurality of practice locations. Internationally, programs also reported rural outcomes with reference to an upper limit of the town’s population, commonly <25,000, <30,000 or <50,000 (19, 56, 101, 106, 116, 156, 165). Frequently, a precise description of the way that graduate rural practice location was determined by programs was not provided (5, 19, 22, 44, 54, 58, 64, 77, 93, 104, 143, 144, 146, 151, 154, 158, 160).

Interest in graduates’ work locations went beyond binary outcomes of rural or metropolitan practice to evaluate the program’s place-based outcomes. These measures included the proportion of graduates practicing in the program’s state/province/prefecture or region (5, 19, 28, 42, 44, 47–49, 56, 64, 77, 79, 93, 110, 113, 128, 129, 131, 143, 151, 154) or in their home state/territory/province/prefecture or other region of origin (48, 64, 77, 94, 106, 133, 134, 155, 165). A small number of programs reported on the contribution of a particular program’s graduates to the region of interest’s workforce (5, 74, 134, 138, 144), including improvements in the availability of junior doctors or physicians in areas of need (65, 76, 93). Involvement in working with under-represented population groups (59, 76, 79, 100, 117, 119, 122, 159) or in underserved areas (5, 43, 77, 93, 100, 103, 110, 119, 141, 156) was also reported (Table 5).

The specialty and context of medical graduates’ practice were commonly evaluated, with a particular focus on family medicine (5, 20, 21, 44, 49, 56, 74, 77, 79, 87, 102, 103, 106, 122, 131, 135, 138, 142, 144, 151, 154, 156, 157, 160), primary care (20, 21, 44, 48, 77, 94, 102, 110, 154, 156, 157) or generalist practice (78, 79, 110, 116, 118, 123), often linked with the greatest workforce needs of the communities being served.

Programs evaluated the impact of widening access to HPE initiatives by investigating the presence of under-represented population groups in their student cohort (Table 5). Programs reported on the presence of rural background students in their cohort (21, 22, 39, 75, 79, 97, 99, 105, 106, 109, 114, 135, 141, 144), and on the presence of students from the program’s defined region (21, 28, 39, 47, 48, 64, 85–89, 91, 108, 109, 114, 151). Other cohort demographics reported included socio-economic status (75, 118, 141), educational background (75, 94, 141), Aboriginal and Francophone student representation (28, 37, 79, 88, 99, 144, 151), and self-identification with an underserved population group (59, 75, 100, 141).

There was limited investigation of the outcomes for the communities in which programs were being delivered. Outcomes evaluated included the correlation of student projects with community needs (21, 93), economic impacts (151, 157) and physician availability (65, 76, 93). There was also a notable absence of published findings regarding the experiences of students and graduates participating in these programs.

## Discussion

Place-based programs identified in this review varied significantly in their geographical scale and design, in part due to the inclusion criteria that allowed for programs up to large state or provincial level in size and the inclusion of a broad range of rural training models. The predominance of medicine in the included programs likely reflects the greater volume of published literature on medical graduate workforce outcomes, which in some countries such as Australia, is a program funding requirement (11).

Despite this heterogeneity and the contextualisation within their global settings, programs were found to be characterized by three common features. Firstly, a comprehensive program design linking targeted student recruitment, rural training and evaluation of relevant outcomes, secondly, a focus on widening access to HPE opportunities, and thirdly, a community-engaged approach. These features align closely with key principles of social accountability, suggesting synergies between social accountability and place-based approaches (29).

### Comprehensive place-based program design

Comprehensive program design refers to the use of multiple interventions or bundled strategies to achieve a program’s goals (1, 6, 20, 21). Programs demonstrated a comprehensive approach to achieving their place-based workforce goals, through targeted recruitment strategies and pre-entry programs, purposeful admission processes and requirements, recurrent or extended training experiences in rural communities and evaluation of outcomes designed to assess the effectiveness of these strategies.

Approaches to student recruitment were heterogenous, illustrating both the lack of a ‘gold standard’ approach and the need for this to be contextualized for each program’s setting. Common characteristics of place-based recruitment were the presence of pre-entry programs that facilitated early connection with local students, strategies to preferentially admit rural students from the region through adjusted admission requirements, and the involvement of the local community in the recruitment process. For medical programs, attributes commonly assessed through a suite of admission requirements included academic capability, rural commitment and often primary care career intentions. These strategies demonstrated that programs were interested not only in rural outcomes, but also in the development of a generalist workforce that would meet the primary healthcare needs of rural communities.

The variation in the geographical scale at which place-based recruitment and training occurred calls for reflection on ideal or preferred approaches. Large state or provincial programs often recruited broadly from their rural region and required students to relocate on more than one occasion for rural training experiences (Table 4, Types 1 and 2), some explicitly valuing the variety of exposure this provided. Other programs had clear mechanisms for students to foster a longitudinal relationship with a single rural community, through entirely rural campus-based programs, or distance education (Table 4, Types 3 and 4). Distance education models (Type 4), prominent in nursing and midwifery, prioritized local student recruitment and keeping learners in their home communities throughout training.

These variations in approach may be due to both practical and pedagogical influences. The absence of Type 4 distance education models in medicine may reflect traditional Flexnerian educational approaches with a period of on-campus pre-clinical learning, allowing for exposure to scientific laboratories and anatomical specimens (167). Extended rural placements have become increasingly common for clinical learning in medicine, illustrated by the predominance of this program design in Table 4. Following the impact of the COVID-19 pandemic during 2019–2021, it will be interesting to observe whether there is an increased uptake of blended learning delivery inclusive of distance education for medical programs. Many programs were forced to ‘pivot’ rapidly to online delivery of learning during the pandemic, a disruptive event that may have an ongoing impact on how programs are delivered that is not yet apparent in the literature (168, 169).

The provision of continuity with a rural community throughout training aligns with evidence that extended rural placements are associated with enhanced rural workforce development (6, 18). Extended rural placements allow students to develop and strengthen their rural identity and self-efficacy, participate more fully as members of the clinical team, and develop meaningful relationships within the community (6, 170–172). Studies that have examined the effect of extended rural training within a student’s own rural region of origin have demonstrated even greater workforce benefits (26). Repeated exposure that provides continuity with the same rural community throughout training, including extended periods of placement time in a students’ home rural community, has the potential to be an important defining feature of authentic place-based program design.

An important consideration is that purposeful selection of students, and location of training sites is not always under the control of individual programs but may be subject to the national policy context. For example, in Australia, rural students are equally eligible for government-mandated rural admission quotas at any rural medical program in the country, meaning students often apply broadly and may relocate long distances for their studies. In contrast in Japan and Thailand, admission quotas operate at a provincial or district level, encouraging a more localised place-based approach. Thus, programs may have been constrained by the policy environment in the extent to which they were able to adopt a place-based approach to student selection and training.

The diversity of outcomes evaluated by programs was perhaps unsurprising given the heterogeneity of included programs. The proposal in this review of a typology distinguishing four models of rural training potentially allows for future identification and comparison of outcomes for narrower clusters of programs with more similar place-based approaches. The utilization of program logic models is recommended to clarify and visually represent the goals and components of programs and how outcomes being evaluated relate to these goals (173, 174). In this way, evaluation can be more clearly linked to the core purposes of these programs to provide a fit-for-purpose rural workforce for their region. The adoption of a program logic model may also draw attention to the importance of evaluating the experience of students participating in these programs, an area identified as a current gap in the literature. Program logic models prompt the development of outcome goals across short, medium and long-term timeframes which would help to shift attention from early outcomes (such as academic equivalence) to evaluate longer term impacts on rural communities, for example improving access to healthcare and health outcomes for underserved populations. Furthermore, the use of program logic models would provide a consistent framework for place-based program description, whilst allowing for customization and clear identification of local contextual factors and assumptions impacting on program design.

Reporting on workforce outcomes for the region of interest, an important feature of place-based programs, is recommended. Ideally, this should include not only the proportion of program graduates working in the region and in primary care but also the contribution of the program’s graduates to the workforce in the region, including other priority specialties based on community needs. A closer examination of workforce outcomes for a more limited range of programs sharing similar design features is warranted to identify factors that positively contribute to graduates working ‘in place’. Programs are encouraged to utilize and clearly define rurality classification systems to describe their place-based geography and apply these measures consistently to student recruitment, training site locations and the measurement of workforce outcomes. This would assist with transparency of how rurality is defined and applied in all elements of program design and enable comparisons to be made between similar programs (175, 176).

### Widening access

Programs expressed clear goals to train fit-for-purpose healthcare professionals for rural and underserved communities, to improve health outcomes for these communities. This ‘back-end’ purpose, to meet societal needs was, in most programs, also associated with a ‘front-end’ purpose, to widen access to their educational programs for students from these same communities (177). While this secondary purpose to widen access was clearly articulated for some programs, for others it could be inferred through the outcomes of interest that were evaluated, for example, the diversity of the student cohort. This duality of purpose to widen access for underserved population groups *from* the region and train *in and for* the region could be considered an identifying hallmark of PBE programs. It is noteworthy that the scale on which these strategies were applied varied significantly, from small rural provinces to entire states, prompting consideration of possible upper limits of scale at which a program could be described as truly place-based.

Place-based program design was found to address both spatial (geographic) and aspatial (economic, socio-cultural, political) barriers to program participation (178). Spatial barriers were addressed by the location of rural learning sites, either through the establishment of new campuses or through de-centralized, distributed learning models. Aspatial barriers, such as financial disadvantage, were also addressed by programs that allowed students to complete their course without the expense involved in relocation, or through the provision of financial assistance. Socio-cultural barriers were addressed by many programs through widening access initiatives in student recruitment, relevant to each program’s context.

### Community engagement

Community engagement is a feature of social accountability that involves authentic interdependent partnerships between health services and academic institutions, respects the knowledge and experience of rural communities and gives them a voice in the selection and training of students (179, 180). This was evident through rural communities being given agency in student selection, with their level of involvement varying from responsibility for nominating students for consideration for program entry (62, 103), to more collaborative involvement as stakeholder representatives on admissions committees (20, 44). Rural communities were also engaged in student training through distributed educational models, often through the provision of clinical supervision. Community engagement was further evident through pre-entry recruitment programs, for example, partnerships with local rural high schools (92, 94, 96).

In terms of program outcomes for communities, measures such as economic impact and healthcare access were reported by a small number of programs. However, there was limited investigation of the broader experiences of communities in which programs were based. This is an important area for future evaluation, given the central role of the community in PBE. Sustainable rural pre-registration HPE programs require collaborative, symbiotic partnerships with health services and communities that enable mutual benefit (181, 182). Evaluation of program outcomes with this focus would recognize the investment of rural communities and provide examples of best practice in this field.

### Limitations

As data for this review were extracted from the peer-reviewed literature only, there may be other place-based pre-registration HPE programs that have not been included. Database search terms were designed to capture a range of terminology and concepts that may be used to describe place-based approaches, however the primary usage of PBE terminology in Western countries may mean that alternative terms used to describe programs adopting similar approaches may not have been captured. A further limitation of this study, with its focus on program design, was the absence of data extraction on rural/place focussed curriculum content and how this may contribute to building and reinforcing place connection. Overall, there was insufficient curricular detail to assess this important aspect of a place-based approach adequately. This knowledge gap could be addressed through a literature review on a narrower subset of place-based pre-registration HPE programs, to explore current practice in this area and how place-based curriculum is being incorporated.

The absence or heterogeneity of rural definitions and classification systems hindered comparisons and contextualisation of programs. This issue has been noted in previous systematic reviews on rural workforce outcomes (4, 5, 19, 77, 175). To assist with this, programs should consider providing a clear and specific description of their defined area of focus, as well as information on how rurality is classified in their region. Population density (persons/square km) was one strategy identified to compare international programs that may be helpful to consider as a unifying program statistic (75, 141).

## Conclusion

Through the identification of common design features of place-based pre-registration HPE programs, this review provides a foundation and framework to guide the establishment and evaluation of similar programs. Key considerations for comprehensive place-based program design include: accurately defining the geographical scale of the program, developing a strategy for student recruitment from the target region focused on widening access, provision of continuity with rural communities through longitudinal training experiences, engaging communities in the design and delivery of the program, and the alignment of evaluation plans with the goals of the program and the communities served. There are rich opportunities for further research into each of these areas, to compare and contrast how place-based HPE programs are delivering on these outcomes through their program design.
