# Supplementary material for: Place-based rural health professional pre-registration education programs: a scoping review
Source: Front Med (Lausanne). 2025 Aug 14;12:1546701. doi: 10.3389/fmed.2025.1546701 (PMC12391070; doi:10.3389/fmed.2025.1546701)
Supplement: Supplementary file 2 [file Table_2.DOCX]

**Supplementary file 2. Electronic search results and terms**

| Ebscohost MEDLINE Complete | 1064 |
| --- | --- |
| Ebscohost CINAHL Complete | 540 |
| Ebscohost ERIC | 162 |
| Embase | 264 |
| Scopus | 2185 |
| **Total** | **4215** |
|  |  |

Database searches conducted on 4 Oct 2023.

**Database: EBSCOhost Medline Complete**

| **Query** | **Search terms** |
| --- | --- |
| S1 | (MH "Students, Health Occupations+") |
| S2 | (MH "Education, Medical, Undergraduate") |
| S3 | (MH "Education, Dental+") |
| S4 | (MH "Education, Pharmacy+") |
| S5 | (MH "Education, Nursing+") |
| S6 | (MH "Schools, Medical") OR (MH "Schools, Nursing") OR OR (MH "Schools, Pharmacy") OR (MH "Schools, Dental") |
| S7 | AB ("medical student*" OR "nursing student*" OR "midwif* student*" OR "dent* student*" OR “allied health student*" OR "pharmac* student*" OR "physiotherap* student*" OR “physical therapy student” OR "speech path* student*" OR "speech language therap* student*" OR "speech language patholog* student*" OR "speech therap* student*" OR "dietitian* student*" OR "nutrition* student*" OR "Aboriginal health worker student*" OR "occupational therap* student*" OR "podiatr* student*" OR "social work student*" OR "audiolog* student*" OR "chiropract* student*" OR "psycholog* student*" OR "exercise physiolog* student*" OR "medical imag* student*" OR “radiog* student*" OR "medical radiation student*" OR “optom* student*” OR “paramedic* student*” OR “osteopath* student*”) OR TI ("medical student*" OR "nursing student*" OR "midwif* student*" OR "dent* student*" OR “allied health student*" OR "pharmac* student*" OR "physiotherap* student*" OR “physical therapy student” OR "speech path* student*" OR "speech language therap* student*" OR "speech language patholog* student*" OR "speech therap* student*" OR "dietitian* student*" OR "nutrition* student*" OR "Aboriginal health worker student*" OR "occupational therap* student*" OR "podiatr* student*" OR "social work student*" OR "audiolog* student*" OR "chiropract* student*" OR "psycholog* student*" OR "exercise physiolog* student*" OR "medical imag* student*" OR “radiog* student*" OR "medical radiation student*" OR “optom* student*” OR “paramedic* student*” OR “osteopath* student*”) |
| S8 | AB ("medical education" OR "nursing education" OR "midwif* education” OR "dent* education" OR “allied health education” OR "pharmac* education" OR "physiotherap* education" OR “physical therapy education” OR "speech path* education" OR "speech language therap* education" OR "speech language patholog* education" OR "speech therap* education" OR "dietitian* education" OR "nutrition* education" OR "Aboriginal health worker education" OR "occupational therap* education" OR "podiatr* education" OR "social work education" OR "audiolog* education" OR "chiropract* education" OR "psycholog* education” OR "exercise physiolog* education" OR "medical imag* education" OR “radiog* education" OR "medical radiation education" OR “optom* education” OR “paramedic* education” OR “osteopath* education”) OR TI ("medical education" OR "nursing education" OR "midwif* education” OR "dent* education" OR “allied health education” OR "pharmac* education" OR "physiotherap* education" OR “physical therapy education” OR "speech path* education" OR "speech language therap* education" OR "speech language patholog* education" OR "speech therap* education" OR "dietitian* education" OR "nutrition* education" OR "Aboriginal health worker education" OR "occupational therap* education" OR "podiatr* education" OR "social work education" OR "audiolog* education" OR "chiropract* education" OR "psycholog* education” OR "exercise physiolog* education" OR "medical imag* education" OR “radiog* education" OR "medical radiation education" OR “optom* education” OR “paramedic* education” OR “osteopath* education”) |
| S9 | AB (medical N2 school* OR nursing N2 school* OR midwif* N2 school* OR dent* N2 school* OR allied health N2 school* OR pharmac* N2 school* OR physiotherap* N2 school* OR physical therapy N2 school OR speech path* N2 school* OR speech language therap* N2 school* OR speech language patholog* N2 school* OR speech therap* N2 school* OR dietitian* N2 school* OR nutrition* N2 school* OR Aboriginal health worker N2 school* OR occupational therap* N2 school* OR podiatr* N2 school* OR social work N2 school* OR audiolog* N2 school* OR chiropract* N2 school* OR psycholog* N2 school* OR exercise physiolog* N2 school* OR medical imag* N2 school* OR radiog* N2 school* OR medical radiation N2 school* OR optom* N2 school* OR paramedic* N2 school* OR “osteopath N2 school*) OR TI (medical N2 school* OR nursing N2 school* OR midwif* N2 school* OR dent* N2 school* OR allied health N2 school* OR pharmac* N2 school* OR physiotherap* N2 school* OR physical therapy N2 school OR speech path* N2 school* OR speech language therap* N2 school* OR speech language patholog* N2 school* OR speech therap* N2 school* OR dietitian* N2 school* OR "nutrition* N2 school* OR Aboriginal health worker N2 school* OR occupational therap* N2 school* OR podiatr* N2 school* OR social work N2 school* OR audiolog* N2 school* OR chiropract* N2 school* OR psycholog* N2 school* OR exercise physiolog* N2 school* OR medical imag* N2 school* OR radiog* N2 school* OR medical radiation N2 school* OR optom* N2 school* OR paramedic* N2 school* OR “osteopath N2 school*) |
| S10 | S1 OR S2 OR S3 OR S4 OR S5 OR S6 OR S7 OR S8 OR S9 |
| S11 | AB (rural OR regional OR remote OR "non-metropolitan" OR "rural area*" ) OR TI ( rural OR regional OR remote OR "non-metropolitan" OR "rural area*" ) |
| S12 | AB ( "grow* your own" OR "grow* our own" ) OR TI ( "grow* your own" OR "grow* our own" ) |
| S13 | AB ( selection OR recruitment OR admission ) OR TI ( selection OR recruitment OR admission ) |
| S14 | AB ( "training pathway" OR "training pipeline" ) OR TI ( "training pathway" OR "training pipeline" ) |
| S15 | AB ( “rural origin” OR “rural background” OR “rural student*” ) OR TI ( “rural origin” OR “rural background” OR “rural student*” ) |
| S16 | TI ( "rural community based education" OR "rural community based medical education" ) OR AB ( "rural community based education" OR "rural community based medical education" ) |
| S17 | TI "place based education" OR AB "place based education" |
| S18 | TI ( "social accountability" OR "socially accountable" OR “social mission” ) OR AB ( "social accountability" OR "socially accountable" OR “social mission” ) |
| S19 | TI ( "rural immersion" OR "rural training" ) OR AB ( "rural immersion" OR "rural training" ) |
| S20 | S12 OR S13 OR S14 OR S15 OR S16 OR S17 OR S18 OR S19 |
| S21 | S10 AND S11 AND S20 (limited to human and English language) |

**Database: EBSCOhost CINAHL Complete**

| **Query** | **Search terms** |
| --- | --- |
| S1 | (MH "Students, Health Occupations+") |
| S2 | (MH "Education, Medical+") OR (MH "Education, Allied Health+") OR (MH "Education, Associate+") OR (MH "Education, Baccalaureate+") OR (MH "Education, Chiropractic") OR (MH "Education, Dental") OR (MH "Education, Diploma Programs+") OR (MH "Education, Midwifery") OR (MH "Education, Nursing+") OR (MH "Education, Pharmacy") OR (MH "Education, Podiatry") OR (MH "Education, Theory-Based+") OR (MH "Entry Into Practice") |
| S3 | (MH "Schools, Health Occupations+") |
| S4 | AB ("medical student*" OR "nursing student*" OR "midwif* student*" OR "dent* student*" OR "allied health student*" OR "pharmac* student*" OR "physiotherap* student*" OR "physical therapy student" OR "speech path* student*" OR "speech language therap* student*" OR "speech language patholog* student*" OR "speech therap* student*" OR "dietitian* student*" OR "nutrition* student*" OR "Aboriginal health worker student*" OR "occupational therap* student*" OR "podiatr* student*" OR "social work student*" OR "audiolog* student*" OR "chiropract* student*" OR "psycholog* student*" OR "exercise physiolog* student*" OR "medical imag* student*" OR “radiog* student*" OR "medical radiation student*" OR “optom* student*” OR “paramedic* student*" OR “osteopath* student*”) OR TI ("medical student*" OR "nursing student*" OR "midwif* student*" OR "dent* student*" OR “allied health student*" OR "pharmac* student*" OR "physiotherap* student*" OR "physical therapy student" OR "speech path* student*" OR "speech language therap* student*" OR "speech language patholog* student*" OR "speech therap* student*" OR "dietitian* student*" OR "nutrition* student*" OR "Aboriginal health worker student*" OR "occupational therap* student*" OR "podiatr* student*" OR "social work student*" OR "audiolog* student*" OR "chiropract* student*" OR "psycholog* student*" OR "exercise physiolog* student*" OR "medical imag* student*" OR "radiog* student*" OR "medical radiation student*" OR “optom* student*” OR “paramedic* student*” OR “osteopath* student*”) |
| S5 | AB ("medical education" OR "nursing education" OR "midwif* education” OR "dent* education" OR "allied health education” OR "pharmac* education" OR "physiotherap* education" OR "physical therapy education" OR "speech path* education" OR "speech language therap* education" OR "speech language patholog* education" OR "speech therap* education" OR "dietitian* education" OR "nutrition* education" OR "Aboriginal health worker education" OR "occupational therap* education" OR "podiatr* education" OR "social work education" OR "audiolog* education" OR "chiropract* education" OR "psycholog* education” OR "exercise physiolog* education" OR "medical imag* education" OR "radiog* education" OR "medical radiation education" OR “optom* education” OR “paramedic* education” OR “osteopath* education”) OR TI ("medical education" OR "nursing education" OR "midwif* education” OR "dent* education" OR "allied health education” OR "pharmac* education" OR "physiotherap* education" OR "physical therapy education" OR "speech path* education" OR "speech language therap* education" OR "speech language patholog* education" OR "speech therap* education" OR "dietitian* education" OR "nutrition* education" OR "Aboriginal health worker education" OR "occupational therap* education" OR "podiatr* education" OR "social work education" OR "audiolog* education" OR "chiropract* education" OR "psycholog* education” OR "exercise physiolog* education" OR "medical imag* education" OR "radiog* education" OR "medical radiation education" OR “optom* education” OR “paramedic* education" OR “osteopath* education”) |
| S6 | AB (medical N2 school* OR nursing N2 school* OR midwif* N2 school* OR dent* N2 school* OR allied health N2 school* OR pharmac* N2 school* OR physiotherap* N2 school* OR "physical therapy N2 school" OR speech path* N2 school* OR speech language therap* N2 school* OR speech language patholog* N2 school* OR speech therap* N2 school* OR dietitian* N2 school* OR nutrition* N2 school* OR Aboriginal health worker N2 school* OR occupational therap* N2 school* OR podiatr* N2 school* OR social work N2 school* OR audiolog* N2 school* OR chiropract* N2 school* OR psycholog* N2 school* OR exercise physiolog* N2 school* OR medical imag* N2 school* OR radiog* N2 school* OR medical radiation N2 school* OR optom* N2 school* OR paramedic* N2 school* OR “osteopath N2 school*) OR TI (medical N2 school* OR nursing N2 school* OR midwif* N2 school* OR dent* N2 school* OR allied health N2 school* OR pharmac* N2 school* OR physiotherap* N2 school* "physical therapy N2 school" OR speech path* N2 school* OR speech language therap* N2 school* OR speech language patholog* N2 school* OR speech therap* N2 school* OR dietitian* N2 school* OR "nutrition* N2 school* OR Aboriginal health worker N2 school* OR occupational therap* N2 school* OR podiatr* N2 school* OR social work N2 school* OR audiolog* N2 school* OR chiropract* N2 school* OR psycholog* N2 school* OR exercise physiolog* N2 school* OR medical imag* N2 school* OR radiog* N2 school* OR medical radiation N2 school* OR optom* N2 school* OR paramedic* N2 school* OR “osteopath N2 school*) |
| S7 | S1 OR S2 OR S3 OR S4 OR S5 OR S6 |
| S8 | AB ( "rural origin" OR "rural background" OR "rural student*" ) OR TI ( "rural origin" OR "rural background" OR "rural student*") |
| S9 | AB ( "training pathway" OR "training pipeline" ) OR TI ( "training pathway" OR "training pipeline" ) |
| S10 | AB ( recruitment OR selection OR admission ) OR TI ( recruitment OR selection OR admission ) |
| S11 | TI ( "grow* your own" OR "grow* our own" ) OR AB ( "grow* your own" OR "grow* our own" ) |
| S12 | TI ( "social accountability" OR "socially accountable" OR "social mission" ) OR AB ( "social accountability" OR "socially accountable" OR "social mission" ) |
| S13 | TI "place-based education" OR AB "place-based education" |
| S14 | TI ( "rural community based education" OR "rural community based medical education" ) OR AB ( "rural community based education" OR "rural community based medical education" ) |
| S15 | TI ( "rural immersion" OR "rural training" ) OR AB ( "rural immersion" OR "rural training" ) |
| S16 | S8 OR S9 OR S10 OR S11 OR S12 OR S13 OR S14 OR S15 |
| S17 | AB (rural OR regional OR remote OR "non-metropolitan" OR "rural area*" ) OR TI ( rural OR regional OR remote OR "non-metropolitan" OR "rural area*" |
| S18 | S7 AND S16 AND S17 (limited to human and English language) |

**Database: EBSCOhost ERIC**

| **Query** | **Search terms** |
| --- | --- |
| S1 | DE "Medical Students") OR (DE "Nursing Students" |
| S2 | DE "Nursing Education" OR DE "Pharmaceutical Education" OR DE "Allied Health Occupations Education" |
| S3 | DE "Medical Schools" OR DE "Dental Schools" |
| S4 | AB ("medical student*" OR "nursing student*" OR "midwif* student*" OR "dent* student*" OR allied health student*" OR "pharmac* student*" OR "physiotherap* student*" OR "speech path* student*" OR "speech language therap* student*" OR "speech language patholog* student*" OR "speech therap* student*" OR "dietitian* student*" OR "nutrition* student*" OR "Aboriginal health worker student*" OR "occupational therap* student*" OR "podiatr* student*" OR "social work student*" OR "audiolog* student*" OR "chiropract* student*" OR "psycholog* student*" OR "exercise physiolog* student*" OR "medical imag* student*" OR “radiog* student*" OR "medical radiation student*" OR “optom* student*” OR “paramedic* student* OR “osteopath* student*”) OR TI ("medical student*" OR "nursing student*" OR "midwif* student*" OR "dent* student*" OR “allied health student*" OR "pharmac* student*" OR "physiotherap* student*" OR "speech path* student*" OR "speech language therap* student*" OR "speech language patholog* student*" OR "speech therap* student*" OR "dietitian* student*" OR "nutrition* student*" OR "Aboriginal health worker student*" OR "occupational therap* student*" OR "podiatr* student*" OR "social work student*" OR "audiolog* student*" OR "chiropract* student*" OR "psycholog* student*" OR "exercise physiolog* student*" OR "medical imag* student*" OR radiog* student*" OR "medical radiation student*" OR “optom* student*” OR “paramedic* student*” OR “osteopath* student*”) |
| S5 | AB ("medical education" OR "nursing education" OR "midwif* education” OR "dent* education" OR allied health education” OR "pharmac* education" OR "physiotherap* education" OR "speech path* education" OR "speech language therap* education" OR "speech language patholog* education" OR "speech therap* education" OR "dietitian* education" OR "nutrition* education" OR "Aboriginal health worker education" OR "occupational therap* education" OR "podiatr* education" OR "social work education" OR "audiolog* education" OR "chiropract* education " OR "psycholog* education” OR "exercise physiolog* education" OR "medical imag* education" OR radiog* education" OR "medical radiation education" OR “optom* education” OR “paramedic* education” OR “osteopath* education”) OR TI ("medical education" OR "nursing education" OR "midwif* education” OR "dent* education" OR allied health education” OR "pharmac* education" OR "physiotherap* education" OR "speech path* education" OR "speech language therap* education" OR "speech language patholog* education" OR "speech therap* education" OR "dietitian* education" OR "nutrition* education" OR "Aboriginal health worker education" OR "occupational therap* education" OR "podiatr* education" OR "social work education" OR "audiolog* education" OR "chiropract* education " OR "psycholog* education” OR "exercise physiolog* education" OR "medical imag* education" OR radiog* education" OR "medical radiation education" OR “optom* education” OR “paramedic* education OR “osteopath* education”) |
| S6 | AB (medical N2 school* OR nursing N2 school* OR midwif* N2 school* OR dent* N2 school* OR allied health N2 school* OR pharmac* N2 school* OR physiotherap* N2 school* OR speech path* N2 school* OR speech language therap* N2 school* OR speech language patholog* N2 school* OR speech therap* N2 school* OR dietitian* N2 school* OR nutrition* N2 school* OR Aboriginal health worker N2 school* OR occupational therap* N2 school* OR podiatr* N2 school* OR social work N2 school* OR audiolog* N2 school* OR chiropract* N2 school* OR psycholog* N2 school* OR exercise physiolog* N2 school* OR medical imag* N2 school* OR radiog* N2 school* OR medical radiation N2 school* OR optom* N2 school* OR paramedic* N2 school* OR “osteopath N2 school*) OR TI (medical N2 school* OR nursing N2 school* OR midwif* N2 school* OR dent* N2 school* OR allied health N2 school* OR pharmac* N2 school* OR physiotherap* N2 school* OR speech path* N2 school* OR speech language therap* N2 school* OR speech language patholog* N2 school* OR speech therap* N2 school* OR dietitian* N2 school* OR "nutrition* N2 school* OR Aboriginal health worker N2 school* OR occupational therap* N2 school* OR podiatr* N2 school* OR social work N2 school* OR audiolog* N2 school* OR chiropract* N2 school* OR psycholog* N2 school* OR exercise physiolog* N2 school* OR medical imag* N2 school* OR radiog* N2 school* OR medical radiation N2 school* OR optom* N2 school* OR paramedic* N2 school* OR “osteopath N2 school*) |
| S7 | S1 OR S2 OR S3 OR S4 OR S5 OR S6 |
| S8 | AB ( "rural origin" OR "rural background" OR "rural student*" ) OR TI ( "rural origin" OR "rural background" OR "rural student*") |
|  | TI ( "rural immersion" OR "rural training" ) OR AB ( "rural immersion" OR "rural training" ) |
| S9 | TI "place based education" OR AB "place based education" |
| S10 | TI ( "social accountability" OR "socially accountable" ) OR AB ( "social accountability" OR "socially accountable" ) |
| S11 | TI ( "rural community based education" OR "rural community based medical education" ) OR AB ( "rural community based education" OR "rural community based medical education" ) |
| S12 | TI ("grow* your own" OR "grow* our own") OR AB ("grow* your own" OR "grow* our own") |
| S13 | TI (selection OR recruitment OR admission) OR AB (selection OR recruitment OR admission) |
| S14 | AB ( "training pathway" OR "training pipeline" ) OR TI ( "training pathway" OR "training pipeline" ) |
| S15 | S8 OR S9 OR S10 OR S11 OR S12 OR S13 OR S14 |
| S16 | DE "Rural Education" OR DE "Rural Areas" |
| S17 | TI(rural OR regional OR remote OR "non-metropolitan" OR "rural area*") OR AB(rural OR regional OR remote OR "non-metropolitan" OR "rural area*") |
| S18 | S16 OR S17 |
| S19 | S7 AND S15 AND S18 (limited to English language) |

**Database: Scopus**

((TITLE-ABS-KEY("medical school*" OR "nursing school*" OR "midwif* school*" OR "dent* school*" OR "allied health school*" OR "pharmac* school" OR "physiotherap* school*" OR "physical therapy school" OR "speech path* school*" OR "speech language therap* school*" OR "speech language patholog* school*" OR "speech therap* school*" OR "dietitian* school*" OR "nutrition* school*" OR "Aboriginal health worker school*" OR "occupational therap* school*" OR "podiatr* school*" OR "social work school*" OR "audiolog* school*")) OR (TITLE-ABS-KEY("chiropract* school*" OR "psycholog* school*" OR "exercise physiolog* school*" OR "medical imag* school*" OR "radiog* school*" OR "medical radiation school*" OR "optom* school*" OR "paramedic* school*" OR "osteopath school*")) OR (TITLE-ABS-KEY("medical education" OR "nursing education" OR "midwif* education" OR "dent* education" OR "allied health education" OR "pharmac* education" OR "physiotherap* education" OR "physical therapy education" OR "speech path* education" OR "speech language therap* education" OR "speech language patholog* education" OR "speech therap* education" OR "dietitian* education" OR "nutrition* education" OR "Aboriginal health worker education" OR "occupational therap* education" OR "podiatr* education" OR "social work education" OR "audiolog* education" OR "chiropract* education " OR "psycholog* education" OR "exercise physiolog* education" OR "medical imag* education" OR "radiog* education" OR "medical radiation education" OR "optom* education" OR "paramedic* education" OR "osteopath* education")) OR (TITLE-ABS-KEY("medical student*" OR "nursing student*" OR "midwif* student*" OR "dent* student*" OR "allied health student*" OR "pharmac* student*" OR "physiotherap* student*" OR "physical therapy student" OR "speech path* student*" OR "speech language therap* student*" OR "speech language patholog* student*" OR "speech therap* student*" OR "dietitian* student*" OR "nutrition* student*" OR "Aboriginal health worker student*" OR "occupational therap* student*" OR "podiatr* student*" OR "social work student*" OR "audiolog* student*" OR "chiropract* student*" OR "psycholog* student*" OR "exercise physiolog* student*" OR "medical imag* student*" OR "radiog* student*" OR "medical radiation student*" OR "optom* student*" OR "paramedic* student*" OR "osteopath* student*"))) AND (TITLE-ABS-KEY(rural OR regional OR remote OR "non-metropolitan" OR "rural area*")) AND ((TITLE-ABS-KEY("grow* your own" OR "grow* our own" OR selection OR recruitment OR admission OR "training pathway" OR "training pipeline") OR TITLE-ABS-KEY("rural origin" OR "rural background" OR "rural student*" OR "rural community based education" OR "rural community based medical education") OR TITLE-ABS-KEY("place based education" OR "place-based education") OR TITLE-ABS-KEY("social accountability" OR "socially accountable" OR "social mission" OR "rural immersion" OR "rural training"))) AND ( LIMIT-TO ( LANGUAGE,"English" ) )

**Database: Embase**

| **Query** | **Search terms** |
| --- | --- |
| #1 | 'medical student':ti,ab,kw OR 'nursing student':ti,ab,kw OR 'dental student':ti,ab,kw OR 'allied health student':ti,ab,kw OR 'audiology student':ti,ab,kw OR 'chiropractic student':ti,ab,kw OR 'dietetics student':ti,ab,kw OR 'midwifery student':ti,ab,kw OR 'occupational therapy student':ti,ab,kw OR 'pharmacy student':ti,ab,kw OR 'physical therapy student':ti,ab,kw OR 'medical education':ti,ab,kw OR 'nursing education':ti,ab,kw OR 'dental education':ti,ab,kw OR 'physical therapy education':ti,ab,kw OR 'allied health education':ti,ab,kw OR 'chiropractic education':ti,ab,kw OR 'dietetics education':ti,ab,kw OR 'emergency medical services education':ti,ab,kw OR 'midwifery education':ti,ab,kw OR 'occupational therapy education':ti,ab,kw OR 'podiatry education':ti,ab,kw OR 'medical school':ti,ab,kw OR 'nursing school':ti,ab,kw OR 'dental school':ti,ab,kw |
| #2 | 'medical student*':ti,ab,kw OR 'nursing student*':ti,ab,kw OR 'midwif* student*':ti,ab,kw OR 'dent* student*':ti,ab,kw OR 'allied health student*':ti,ab,kw OR 'pharmac* student*':ti,ab,kw OR 'physiotherap* student*':ti,ab,kw OR 'physical therapy student':ti,ab,kw OR 'speech path* student*':ti,ab,kw OR 'speech language therap* student*':ti,ab,kw OR 'speech language patholog* student*':ti,ab,kw OR 'speech therap* student*':ti,ab,kw OR 'dietitian* student*':ti,ab,kw OR 'nutrition* student*':ti,ab,kw OR 'aboriginal health worker student*':ti,ab,kw OR 'occupational therap* student*':ti,ab,kw OR 'podiatr* student*':ti,ab,kw OR 'social work student*':ti,ab,kw OR 'audiolog* student*':ti,ab,kw OR 'chiropract* student*':ti,ab,kw OR 'psycholog* student*':ti,ab,kw OR 'exercise physiolog* student*':ti,ab,kw OR 'medical imag* student*':ti,ab,kw OR 'radiog* student*':ti,ab,kw OR 'medical radiation student*':ti,ab,kw OR 'optom* student*':ti,ab,kw OR 'paramedic* student*':ti,ab,kw OR 'osteopath* student*':ti,ab,kw |
| #3 | 'medical education':ti,ab,kw OR 'nursing education':ti,ab,kw OR 'midwif* education':ti,ab,kw OR 'dent* education':ti,ab,kw OR 'allied health education':ti,ab,kw OR 'pharmac* education' OR 'physiotherap* education':ti,ab,kw OR 'physical therapy education':ti,ab,kw OR 'speech path* education':ti,ab,kw OR 'speech language therap* education':ti,ab,kw OR 'speech language patholog* education':ti,ab,kw OR 'speech therap* education':ti,ab,kw OR 'dietitian* education':ti,ab,kw OR 'nutrition* education':ti,ab,kw OR 'aboriginal health worker education':ti,ab,kw OR 'occupational therap* education':ti,ab,kw OR 'podiatr* education':ti,ab,kw OR 'social work education':ti,ab,kw OR 'audiolog* education':ti,ab,kw OR 'chiropract* education':ti,ab,kw OR 'psycholog* education':ti,ab,kw OR 'exercise physiolog* education':ti,ab,kw OR 'medical imag* education':ti,ab,kw OR 'radiog* education':ti,ab,kw OR 'medical radiation education':ti,ab,kw OR 'optom* education':ti,ab,kw OR 'paramedic* education':ti,ab,kw OR 'osteopath* education':ti,ab,kw |
| #4 | 'medical school*':ti,ab,kw OR 'nursing school*':ti,ab,kw OR 'midwif* school*':ti,ab,kw OR 'dent* school*':ti,ab,kw OR 'allied health school*':ti,ab,kw OR 'pharmac* school*':ti,ab,kw OR 'physiotherap* school*':ti,ab,kw OR 'physical therapy school':ti,ab,kw OR 'speech path* school*':ti,ab,kw OR 'speech language therap* school*':ti,ab,kw OR 'speech language patholog* school*':ti,ab,kw OR 'speech therap* school*':ti,ab,kw OR 'dietitian* school*':ti,ab,kw OR 'nutrition* school*':ti,ab,kw OR 'aboriginal health worker school*':ti,ab,kw OR 'occupational therap* school*':ti,ab,kw OR 'podiatr* school*':ti,ab,kw OR 'social work school*':ti,ab,kw OR 'audiolog* school*':ti,ab,kw OR 'chiropract* school*':ti,ab,kw OR 'psycholog* school*':ti,ab,kw OR 'exercise physiolog* school*':ti,ab,kw OR 'medical imag* school*':ti,ab,kw OR 'radiog* school*':ti,ab,kw OR 'medical radiation school*':ti,ab,kw OR 'optom* school*':ti,ab,kw OR 'paramedic* school*':ti,ab,kw OR 'osteopath* school*':ti,ab,kw |
| #5 | #1 OR #2 OR #3 OR #4 |
| #6 | 'grow* your own':ti,ab,kw OR 'grow* our own':ti,ab,kw |
| #7 | selection:ti,ab,kw OR recruitment:ti,ab,kw OR admission:ti,ab,kw |
| #8 | 'training pathway':ti,ab,kw OR 'training pipeline':ti,ab,kw |
| #9 | 'rural origin':ti,ab,kw OR 'rural background':ti,ab,kw OR 'rural student*':ti,ab,kw |
| #10 | 'rural community based education':ti,ab,kw OR 'rural community based medical education':ti,ab,kw |
| #11 | 'place-based education':ti,ab,kw |
| #12 | 'social accountability':ti,ab,kw OR 'socially accountable':ti,ab,kw OR 'social mission':ti,ab,kw |
| #13 | 'rural immersion':ti,ab,kw OR 'rural training':ti,ab,kw |
| #14 | #6 OR #7 OR #8 OR #9 OR #10 OR #11 OR #12 OR #13 |
| #15 | rural:ti,ab,kw OR regional:ti,ab,kw OR remote:ti,ab,kw OR 'non-metropolitan':ti,ab,kw OR 'rural area*':ti,ab,kw |
| #16 | #5 AND #14 AND #15 |
| #17 | #16 AND [embase]/lim NOT ([embase]/lim AND [medline]/lim) AND [humans]/lim AND [english]/lim |
